# Evaluating cell culture reliability in pediatric brain tumor primary cells through DNA methylation profiling

**DOI:** 10.1038/s41698-024-00578-x

**Published:** 2024-04-18

**Authors:** Lucia Pedace, Simone Pizzi, Luana Abballe, Maria Vinci, Celeste Antonacci, Sara Patrizi, Claudia Nardini, Francesca Del Bufalo, Sabrina Rossi, Giulia Pericoli, Francesca Gianno, Zein Mersini Besharat, Luca Tiberi, Angela Mastronuzzi, Elisabetta Ferretti, Marco Tartaglia, Franco Locatelli, Andrea Ciolfi, Evelina Miele

**Affiliations:** 1 Onco-Hematology, Cell Therapy, Gene Therapies and Hemopoietic Transplant, Bambino Gesù Children’s Hospital, IRCCS, Rome, Italy; 2 Molecular Genetics and Functional Genomics, Bambino Gesù Children’s Hospital, IRCCS, 00146 Rome, Italy; 3 Pathology Unit, Department of Laboratories, Bambino Gesù Children’s Hospital, IRCCS, Rome, Italy; 4 Department of Radiological, Oncological and Anatomic Pathology, Sapienza University, Rome, Italy; 5 Department of Experimental Medicine, “Sapienza” University, 00161 Rome, Italy; 6 Armenise-Harvard Laboratory of Brain Disorders and Cancer, Department of Cellular, Computational and Integrative Biology (CIBIO), University of Trento, Trento, Italy

**Keywords:** Cancer models, CNS cancer

## Abstract

In vitro models of pediatric brain tumors (pBT) are instrumental for better understanding the mechanisms contributing to oncogenesis and testing new therapies; thus, ideally, they should recapitulate the original tumor. We applied DNA methylation (DNAm) and copy number variation (CNV) profiling to characterize 241 pBT samples, including 155 tumors and 86 pBT-derived cell cultures, considering serum *vs* serum-free conditions, late *vs* early passages, and dimensionality (2D *vs* 3D cultures). We performed a t-SNE classification and identified differentially methylated regions in tumors compared to cell models. Early cell cultures recapitulate the original tumor, but serum media and 2D culturing were demonstrated to significantly contribute to the divergence of DNAm profiles from the parental ones. All divergent cells clustered together acquiring a common deregulated epigenetic signature suggesting a shared selective pressure. We identified a set of hypomethylated genes shared among unfaithful cells converging on response to growth factors and migration pathways, such as signaling cascade activation, tissue organization, and cellular migration. In conclusion, DNAm and CNV are informative tools that should be used to assess the recapitulation of pBT-cells from parental tumors.

## Introduction

Central nervous system (CNS) tumors are among the most common cancers in patients aged 0–14 years^1^. Recent genomic and epigenomic profiling analyses have provided remarkable insights into biology of pediatric brain tumors (pBTs)^2^. Nonetheless, pBTs represent the deadliest childhood cancer worldwide and are associated with high morbidity with the consequent and urgent need for therapeutic advances to improve the outcome and quality of life of affected children^3^.

To this end, scientists take advantage of informative, stable, and faithful model systems, exploring the mechanisms of the disease and testing new targeted therapeutic approaches. Developing and optimizing such systems, including in vitro models, represents a mandatory field of research. Furthermore, the choice of cell culture medium for in vitro pBT biopsy cultures must be taken into consideration, especially for drug screening experiments^4^.

Much research in pBTs biology relies on experiments under stem cell conditions, either as two-dimensional (2D) adherent cultures or as three-dimensional (3D) neurospheres^5–7^. Recently, several groups validated a more complex in vitro culture system of human cerebellar organoids as reliable modeling for CNS studies^8–10^.

Genome-wide DNAm is a stable epigenetic process regulating the diversified gene expression profiles characterizing cells and tissues in multicellular organisms. DNAm profiling is an informative tool that aids in the classification of CNS tumors and patient stratification, favoring a more effective patient’s management^11–13^. It is increasingly incorporated in the diagnostic process of CNS tumors, and it is a powerful tool to confirm their pathological diagnosis^14–16^.

The current classification occurs using the DNAm-based CNS tumor classifier (www.molecularneuropathology.org), which generates a calibrated score, representing the degree of match between the methylation profile of the tumor of interest and predefined methylation classes (MC)^17^. DNAm array data can also be used to evaluate the copy-number variations (CNV), providing a good overview of gross structural alterations in the tumor genome, that result in an abnormal number of copies of one or more genes such as high-level amplifications and deletions^17^. The CNV plot, generated by the classifier, can be easily interpreted, and it is of considerable added value in tumor entities that exhibit characteristic chromosomal aberrations.

Recently, several research groups have provided useful information about epigenetic differences between tissues with matching cell lines derived from them^18–22^. They have shown that the cultured media and cell line passage numbers could affect the epigenetic pattern^23–25^. These analyses provide supporting evidence in favor of using cell lines as in vitro models in cancer research, although they focused on specific cell models and do not give information on the most suitable cell culture method for faithfully reproducing the parental tumor^26–28^.

At late passages, it is generally understood that clonal selection might represent a relevant event; when this occurs, the culture might not be representative of the parental tumoral cell population, so the experiments can generate variable results^27^, leading to the alteration of cell line susceptibility to external insult^29^. Similarly, different choices of media and additives may have a profound effect on the cell phenotype and drive clonal selection, affecting both the cell behavior and loss of intratumoral diversity^30^.

Taking advantage of a large availability of CNS tumor tissues and their matching derived cell lines, and considering the lack of knowledge, we decided to focus on DNAm status and CNV profiling of pBT-derived primary cultures in different conditions to validate their use as an informative and faithful in vitro model system to represent the corresponding tumor tissue.

## Results

### Brain tumor DNAm profiling and classification

**Fig. 1:**
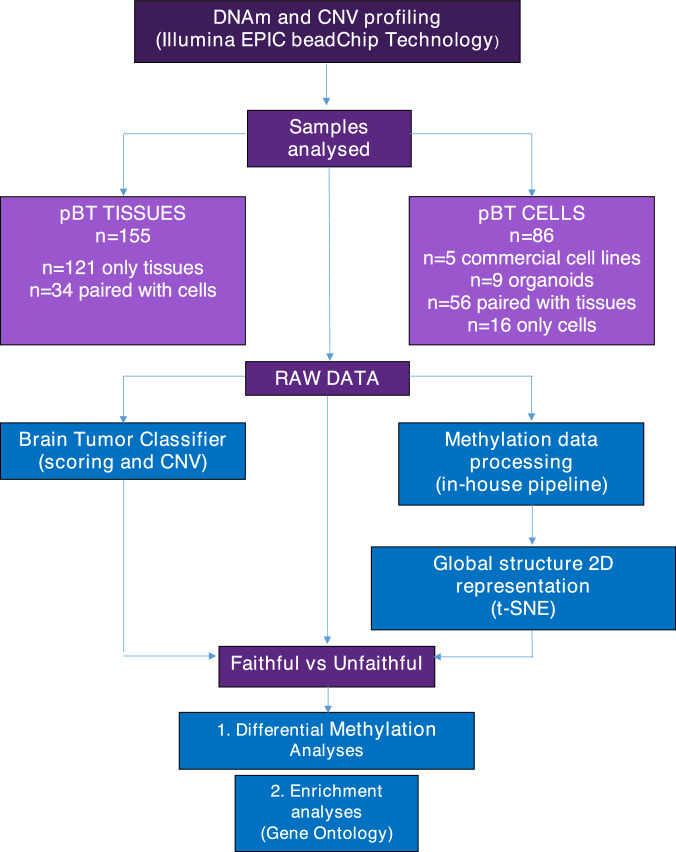
Flowchart of methylation based pBT analysis. The pipeline consists of cells and tumors selection, raw data generation through Illumina EPIC beadChip technology, brain tumor classifier analysis and t-SNE classification, “Faithful vs Unfaithful” definition. Each part is described in the Materials and Methods section. Created by Biorender.com.

We performed DNAm profiling of 241 samples (155 pBT tissues and 86 tumor-derived cell lines) (Fig. 1 and Supplementary Table 1) and compared the results of the brain tumor classifier (v12.5) with the initial histopathological assessment. Considering the tumors cohort, the percentage of matches resulting from the comparison between the two classifications was 99%. Overall, the percentage of match with the 86 cell lines analyzed was 48% (Table 1).

**Table 1:** Entire cohort of 241 pediatric brain tumor samples, including 155 tumors and 86 derived cell cultures, and comparison between the initial histopathological assessment and the results of the brain tumor classifier (v12.5)

Tissues						Cells					
Histopathological assessment	Samples	Paired with cells	Only tissues	Tot	Faithful + Useful (%)	Paired with tissues	Only cells	Organoids	Commercial	Tot	Faithful + Useful (%)
**MB**	**82**	5	56	**61**	61 (100)	8	0	9	4	**21**	15 (71)
**LGG**	**67**	13	32	**45**	43 (95)	20	2	0	0	**22**	3 (14)
**HGG**	**39**	8	15	**23**	23 (100)	15	1	0	1	**17**	5 (29)
**DMG**	**37**	6	7	**13**	13 (100)	11	13	0	0	**24**	16 (67)
**EPN**	**11**	2	7	**9**	9 (100)	2	0	0	0	**2**	2 (100)
**MNG**	**4**	0	4	**4**	4 (100)	0	0	0	0	**0**	0
**TOTAL**	**241**	34	121	**155**	153 (99)	56	16	9	5	**86**	41 (48)

For each pediatric brain tumor category (tissues and cells), the proportion of classifier results that match histopathological data is reported. Faithful: classifier score > 0.84; Useful: classifier score between 0.3 and 0.84.

**Fig. 2:**
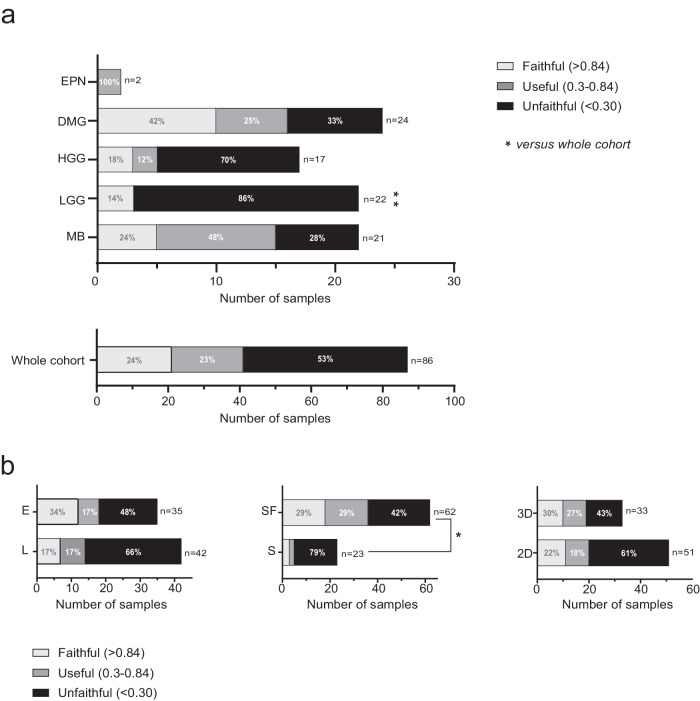
Percentage of match through the brain tumor classifier. **a** Distribution of the scores for whole cohort (bottom panel) and for each tumor category (MB, LGG, HGG, DMG and EPN) (upper panel). Cell cohort is analyzed through the “classifier” and is divided according to methylation score in three classes: (i) unfaithful (samples are not assigned in the MC and/or a score lower than 0.3); (ii) useful (0.3 < score < −0.84) (samples are assigned in the right MC with a score between 0.3 and 0.84); (iii) faithful (>0.84) (samples are assigned in the right MC with a score higher than 0.84). The color intensity of the pie charts reflects the difference in score. Two-tail *p*-value = 0.005 (Fisher’s test) versus the whole cohort. **b** Comparison of cell score distribution for each cell cultures category (culture method, time and dimensionality). *P*-value = 0.0029 (Chi-squared test) refers to Faithful plus Useful vs Unfaithful categories, details in Supplementary Table 3.

We defined the fidelity of the cell cultures compared to the classification scores of the parental pBT according to the classification scores (Fig. 2 and Supplementary Table 1) dividing samples into three groups: (i) unfaithful (score < 0.3), (ii) useful (0.3 < score < 0.84) and (iii) faithful (score > 0.84) samples. Unexpectedly, considering both faithful and useful scores, less than 50% of primary/commercial cell lines and organoids matched the parental pBT classification (Fig. 2a). Specifically, a faithful score was assigned in 24% (5/21) of MB, 14% (3/22) of LGG, 18% (3/17) of HGG, 42% (10/24) of DMG cell cultures, while a useful score was assigned in 48% (10/21) of MB, 12% (2/17) of HGG, 25% (6/24) of DMG, and 100% (2/2) of EPN cell cultures (Fig. 2a).

Primary cell lines that recapitulate paired tumor tissue showed a similar classifier score in the corresponding MC, revealing that they were not markedly impaired in culture system. Half of the cultures were unfaithful and were included, regardless of the tissue of origin, in the meningioma MC (20.9%) with suboptimal raw and calibrated *scores, CIC*-rearranged sarcoma MC (1.2%), or resulted unclassifiable (27.9%) (Supplementary Table 1). Of note, LGG cells were enriched in unfaithful samples compared to the whole cell cohort (*p*-value = 0.005) (Fig. 2a).

To characterize the unfaithful cell lines in more detail, we performed further comparisons based on (i) time of culture (early/late); (ii) culture method (serum/serum free); and (iii) dimensional culture condition (2D vs 3D).

Analyzing cell culture over time, the consistent MC was maintained in a substantial percentage of the “early” cells compared to the “late” ones (*p*-value = 0.1) (Fig. 2b left and Supplementary Table 2).

The evaluation of the impact of culture conditions showed that consistent MC was maintained in a substantial proportion of those grown with serum-free conditions, while a higher proportion of cells maintained in serum cultures diverged in their DNAm profile (*p*-value = 0.029) (Fig. 2b center, and Supplementary Table 2).

Considering the “dimensional culture conditions”, 3D cells were more consistent in the MC than 2D cells even if not reaching a statistical significance (*p*-value = 0.099) (Fig. 2b right and Supplementary Table 2).

The analysis of the DNAm profile of five commercial cell lines demonstrated that three of them maintained the expected MC. Notably, among the four MB commercial cell lines, DAOY, a 2D-serum-late culture, did not classify in any MC by the brain tumor classifier. Similarly, one HGG cell line (KNS42), assessed at late passages and cultured as 2D in the presence of serum, resulted unfaithful. D341 and D283 cells were analyzed as serum-late cultures in semi-suspension (2D/3D). For both, the brain tumor classifier assigned the consistent MC with a useful score. CHLA cells were analyzed as 3D-serum-late culture and classified as medullomyoblastoma with useful score (details in Supplementary Table 1).

The classification scores of 56 tumor tissues with their paired cell lines and the classification scores of 30 cell lines not paired with their tissue of origin are reported in Supplementary Fig. 1.

### Copy number variation analysis

**Fig. 3:**
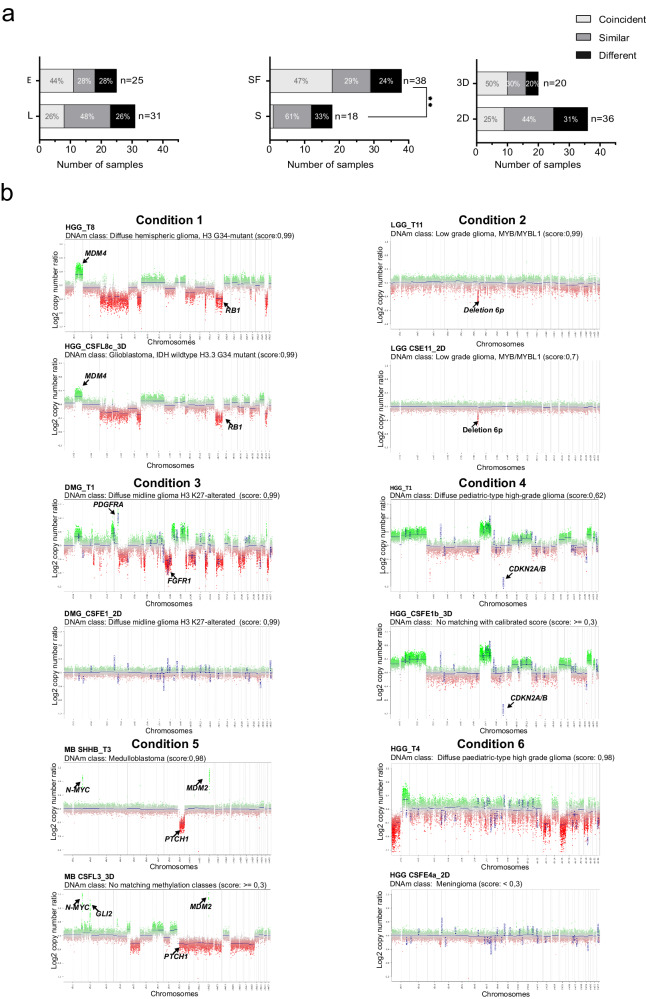
Comparison of Copy Number Variation data in paired primary cells and tissues. **a** The color intensity of the histograms reflects the three categories of similarity between the CNV of each cell line with corresponding tumor, according to the correlation score: coincident (top tertile), similar (middle tertile), and different (lower tertile). *p*-value = 0.005 (Chi-squared test) refers to coincident plus similar *vs* different categories, details in Supplementary Table 4. **b** *Condition 1:* HGG_T8 *versus* HGG_CSFL8c_3D. Both classified as diffuse hemispheric glioma, H3 G34-mutant MC, and conserved the same CNV. *Condition 2:* LGG_T11 *versus* LGG_CSE11_2D. The classifier assigned the sample to the low-grade glioma, *MYB/MYBL1* MC. The CNV plot revealed a 6p deletion in the tumor. The derived cell displayed consistent DNAm levels, along with the 6p deletion. *Condition 3:* DMG_T1 *versus* DMG_CSFE1_2D. Both faithfully classified as diffuse midline glioma H3 K27-alterated. The parental tumor CNV profile exhibited *PDGFRA* amplification, and *FGFR1* deletion, while the cell line lost all genetic alterations. *Condition 4:* HGG_T1 tumor matched the MC of diffuse pediatric-type high-grade glioma, H3-wildtype and IDH-wildtype with a suboptimal classifier score. It displayed a complex CNV. HGG_CSFE1b_3D maintained the same CNV but had no matching MC. *Condition 5:* MB_T3 *versus* MB_CSFL3_3D. MB_CSFL3_3D showed an unfaithful MC and conserved both *MYCN* and *MDM2* amplifications. *Condition 6:* HGG_T4 matched the MC of diffuse pediatric-type high-grade glioma, H3-wildtype and IDH-wildtype with an optimal classifier score. It displayed a complex CNV profile. HGG CSFE4a_2D had no matching MC and lost the same CNV.

To further characterize pBT-derived primary cell lines, we analyzed genome-wide DNAm patterns coupled with structural variants (CNV). We identified coincident, similar or different CNV profiles, comparing the original tumor with respective cell cultures and considering the three different variables (serum/serum free; early/late, 2D *vs* 3D) (Supplementary Table 3). The following statistical analyses were performed grouping coincident and similar profiles in a single category of “maintained” CNV profiles. We observed maintained CNV profiles in a sizable portion of serum-free cell cultures compared to the ones cultured with serum (*p*-value = 0.661). Furthermore, a maintained profile was observed in most of 3D compared to 2D cell cultures (*p*-value = 0.589). Finally, a maintained CNV profile was observed in a similar portion of both “early” and “late” cell cultures (*p*-value = 0.905). Of note, when considering only coincident CNV profiles, we found them significantly increased in serum-free *vs* serum cell cultures (*p*-value = 0.005) (Fig. 3a and Supplementary Table 4).

Through a quantitative analysis (Supplementary Fig. 2a), we observed that cell lines with different CNV tended to have a lower mean number of genome-wide CNVs than their tissue counterparts of the same histology. This difference was statistically significant in DMG (*p*-value = 0.023), HGG (*p*-value = 0.005), LGG (*p*-value = 0.005), and MB (*p*-value = 0.025).

We also plotted the cumulative CNV profiles of tumors, grouped by histology (Supplementary Fig. 2b, panels labeled with “1”), and the respective cell lines with different profiles (Supplementary Fig. 2b, panels labeled with “2”). From this visual comparison, we appreciated that different cells show a flatter profile than their tissue counterparts.

Combining DNAm and CNV data we observed six different conditions between cell cultures and tumors considering maintained or unfaithful DNAm, and coincident, similar or different CNV (Table 2, Supplementary Table 3, and Fig. 3a).

**Table 2:** Different conditions for the pBT tissues with corresponding cell lines combining DNAm and CNV

Condition	DNAm	CNV profile	Observed cases (%)	Representative cases	
				Tissue (T)	Cell line*
**1**	Maintained	Coincident	25	HGG_T8	HGG_CSFL8c_3D
**2**	Maintained	Similar	6	LGG_T11	LGG_CSE11_2D
**3**	Maintained	Different	5	DMG_T1	DMG_CSFE1_2D
**4**	Unfaithful	Coincident	9	HGG_T1	HGG_CSFE1b_3D
**5**	Unfaithful	Similar	33	MB_SHHB_T3	MB_CSFL3_3D
**6**	Unfaithful	Different	22	HGG_T4	HGG_CSFE4a_2D

Tumor tissues (T) and cell lines (C) are named with the tumor acronym and progressive numbers, *Cell lines were named according to the nomenclature below: primary cultures (C), either as serum-free (SF) or serum-supplemented medium (S) and considering early (E) and long (L) passages in culture, in both two-dimensional (2D) and three-dimensional (3D) conditions. Different cell lines method cultures are indicated with italic letters.

*DNAm* DNA methylation, *CNV* copy number variations, *LGG* low-grade gliomas, *HGG* high-grade gliomas, *DMG* diffuse (pontine) midline gliomas H3 K27M-altered, *MB* medulloblastoma.

### DNA methylation data processing and sample clustering

To characterize the genome-wide DNAm profiles of the patient-derived cell lines, all samples were analyzed using an in-house developed pipeline for Illumina EPIC BeadChip data. After applying quality filters (see Methods) 676,852 probes remained for the analysis of the study cohort. Neither PCA nor MDS showed the presence of any outlier sample, and, after normalization, the expected bimodal beta value distribution was observed for all 241 samples. SVD analysis showed no significant component (PC) correlating with technical sources of variation (*i.e*. Material, Slide, Slide position), excluding the occurrence of relevant batch effects (data not shown).

**Fig. 4:**
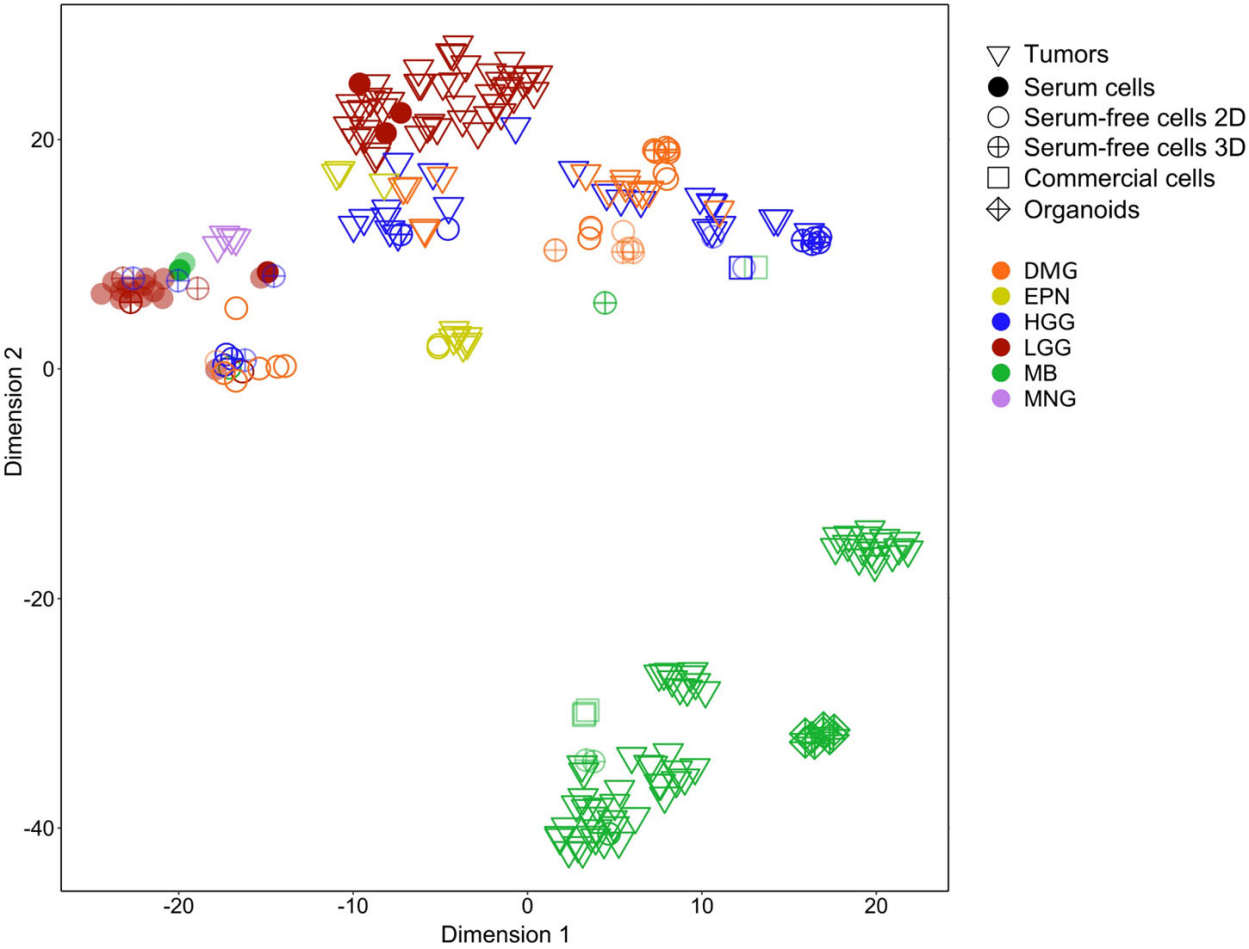
Global structure 2D projection of methylation data. t-SNE analysis of DNAm data of 155 tissues and 86 tumor-derived cell lines. The color has been shaded for those cells whose time in culture is “late”. Points shape was used for distinguishing tumor tissues from cell lines and for specifying both cells culture media and cells culture method.

As shown in Fig. 4 and S3, t-SNE outlined clear segregation among different methylation clusters for most of tumor samples. The analysis showed also a different clustering of the cell lines depending on the culture media and passages. Unsupervised HDBSCAN analysis of the t-SNE projection showed that 220 samples were assigned to 16 distinct and valid clusters, according to DBCV analysis (all clusters with validity indexes > 0.25), whereas 15 were classified as noise points (cluster #0, Supplementary Fig. 3, and Supplementary Table 5).

Among the 8 primary MB-cell lines, 5 displayed a methylation profile not segregating with tissues: two cell lines with serum (”early” and “late”), one “early” cell line serum free-2D, and two “late” serum free-3D. On the other hand, all the engineered group 3 MB organoids were distributed close to the consistent human specimens. By contrast, three of the four commercial MB cell lines (D283, D341, CHLA) clustered near MB tissues, whereas DAOY was found to be unfaithful also in terms of t-SNE location. Interestingly, the HGG cell lines KNS42 clustered together with DAOY and close to HGG tissues.

Three out of the 22 LGG cell lines segregated with the corresponding tissues (all were 2D-” early” and serum cells). On the other hand, 19 LGG cell lines did not cluster with corresponding tumor specimens and were distributed in two different clusters: 2 cell lines were near the cluster of serum free-2D cell lines (cluster #14), 15 were close to meningioma samples (cluster #15), and 2 in the cluster #0 (Supplementary Fig. 3 and Supplementary Fig. 4).

Analysis of the DNAm profiles of the 16 HGG serum-free cell lines (6 cell lines 2D and 10 cell lines 3D) showed that 4 “early” 2D cultures, 1 “early” 3D cultures, and 7 “late” 3D cultures did not cluster with their parental tissues.

Analyzing the 24 DMG primary cultures, within the 18 serum-free, 2D cell lines (12 “early” and 6 “late”), the methylation clustering was maintained by 6 “early” and 4 “late” cell lines, while among the 6 serum-free 3D cell lines, only one “late” cell lines did not segregate with the corresponding tissue.

Lastly, both serum-free “early” cell lines from EPN clustered with the corresponding tissues.

Several divergent cell cultures (*n* = 22) were located close to meningioma samples, and 19 of them classified as a stable cluster (#15) (Supplementary Fig. 3, Supplementary Fig. 4, and Supplementary Table 5).

### Genome-wide differentially methylated regions in cell lines in pBTs

Based on the distribution and relations among samples over the 2D-structure of methylation data, we set up different strategies to identify significant DNAm differences among groups. Since all the divergent cell lines clustered together, we hypothesized a shared selective pressure leading to a common deregulated epigenetic signature. To investigate this assumption, we compared samples that were part of separate HDBSCAN clusters. Hereafter, we will refer to these analyses as DCA1 and DCA2 (DMR cluster analysis 1 and 2).

DCA1 aimed to assess molecular differences underlying all the unfaithful cells (clusters #14 and #15) with respect to most tumor samples and the respective faithful derived cell lines (mainly part of clusters #9, #12, #13) (Supplementary Fig. 3).

DCA2 aimed to investigate molecular differences between unfaithful serum free-2D cell lines (cluster #14) and meningioma-like cells (cluster #15) (Supplementary Fig. 3).

To investigate more specifically the methylation differences between faithful and unfaithful cell cultures, we also defined supervised groups regardless of the HDBSCAN clustering by collecting cells into custom groups based on their fidelity. Further DMR analyses were therefore performed between these groups of cells belonging to the MB, LGG, HGG, and DMG tumor classes to identify specific enriched pathways. Details about test/reference group composition and number of DMRs found for each of the 7 DMR analyses performed are reported in Supplementary Table 6.

An initial gene set enrichment analysis over DMRs was performed to test for both GO terms and KEGG pathway enrichments, and for “Hallmark gene sets” of the Human MSigDB database.

We obtained 32 significant hallmark gene sets for the DCA1 analysis and 25 gene sets for the DCA2 analysis. A mean of 9 enriched gene sets were instead found significant over the MB, LGG, HGG, DMG cell groups (Supplementary Table 7). Among them, the “EPITHELIAL_MESENCHYMAL_TRANSITION” gene set turned out to be significantly enriched in all seven analyses (DCA1, DCA2, MB, LGG, HGG, DMG). Similarly, “MYOGENESIS” was significant in all the groups except for MB. All the significant Hallmark gene sets detected are reported in Supplementary Table 8.

The GO enrichment analysis for the Biological Process allowed us to obtain 1,174 and 658 enriched terms for the DCA1 and DCA2, respectively (Supplementary Table 7). By collecting genes from all significant GO terms of the missMethyl output, 2,945 genes were found, on average, for the four cell type groups and 8917 for the DCA1/2 analyses. In parallel, to focus only on those genes with a high degree of hyper-/hypo- methylation, we associated each DMR with its methylation level (Δβ) so that we could create separated hypo-/hyper-methylated gene lists to be submitted to clusterProfiler. Notably, in the missMethyl output significant hypomethylated genes were in greater number than significant hypermethylated genes (378 average hyper- vs. 539 average hypo-methylated genes; Supplementary Table 7).

**Fig. 5:**
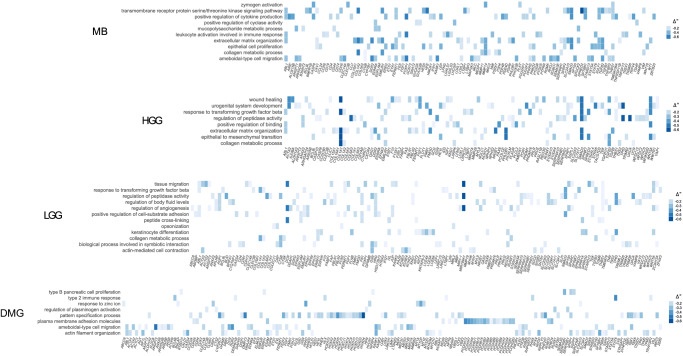
Heatmap plots of Gene Ontology terms enriched in unfaithful cells. Enriched GO terms and related genes reflecting the differential methylation patterns in unfaithful cells of the MB, HGG, LGG, and DMG cell lines. The enrichment analysis shown here was performed with hypomethylated genes that fall in DMRs; for GO terms associated with hypermethylated genes see Supplementary Fig. 5. The ∆β value, obtained from average β-values of CpGs contained in DMRs, was used in place of fold change as a measure of methylation levels.

Moreover, clusterProfiler detected a median of 154 enriched GO terms in the analysis of hypomethylated genes, and only 33 with the hypermethylated genes. Only the LGG enrichment analysis showed opposite results, i.e., 388 GO terms resulted from hypermethylated genes (764) and 92 terms from hypomethylated genes (611). This difference was no longer observed after the semantic similarity analysis (Fig. 5 and Supplementary Fig. 5).

We also investigated DNAm levels for genes belonging to significantly enriched GOs, finding a marked unbalance towards hypomethylation in the MB, HGG, and DMG analyses (median: 271 hypomethylated *vs* 57 hypermethylated). This was not true for clusters #14 and #15 taken together (DCA1), as well as for the LGG cell group in which hypermethylated genes were in greater number (376 hyper- vs. 259 hypo-methylated genes). All these data are collected in Supplementary Table 7.

**Fig. 6:**
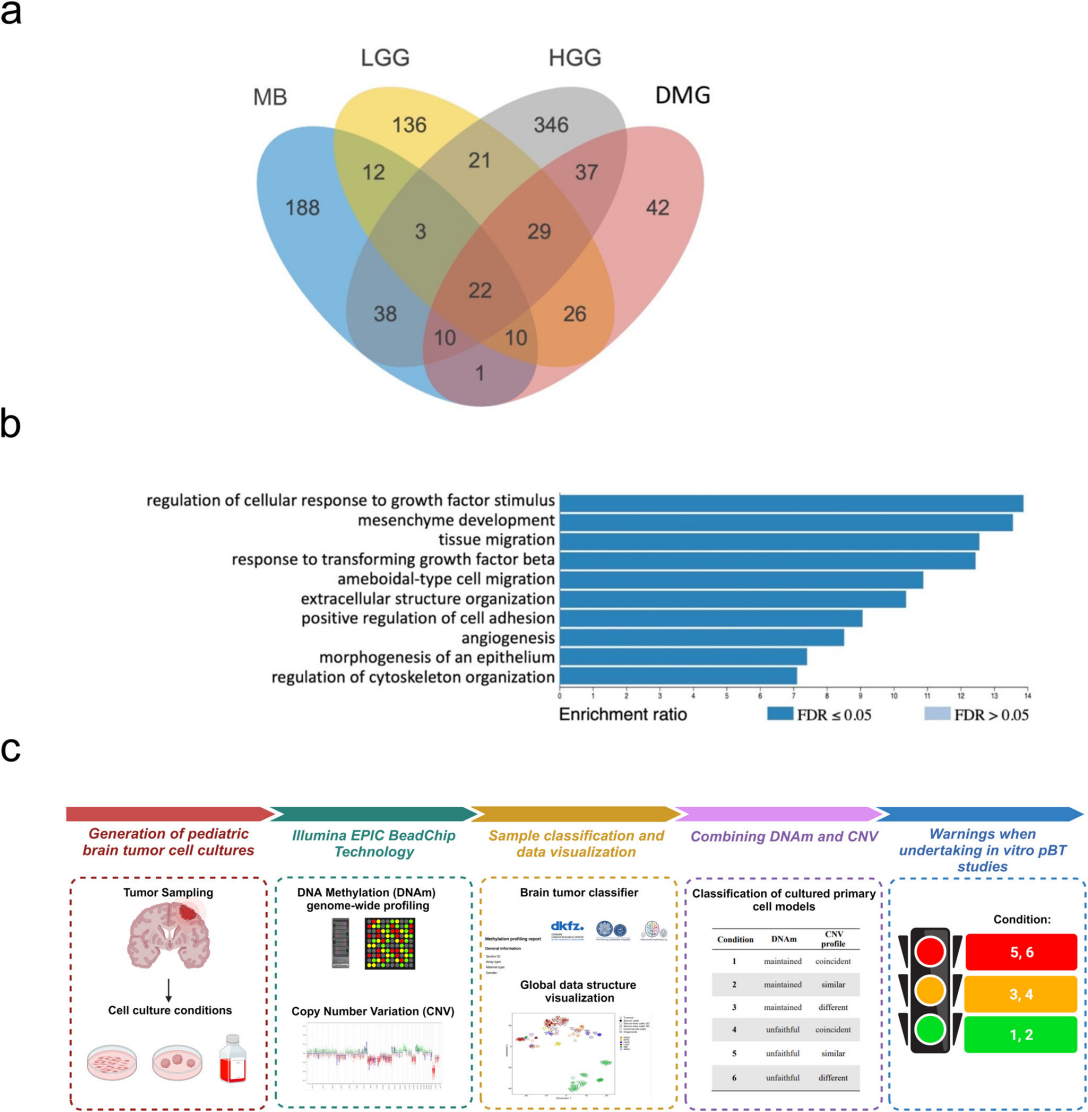
Overlap of hypomethylated genes among the tumor-derived cell lines and final model. **a** Relations among the sets of hypomethylated genes of the 4 types of unfaithful tumor-derived cells and (**b**) GO terms resulting from Over-Representation Analysis over the 22 genes^*^ shared among the 4 sets (http://www.webgestalt.org). ^*^ABL1, ACTG2, BCL9L, CALD1, CD4, CLEC14A, CLEC3B, COL16A1, COL1A2, DDR2, EDNRA, EMILIN1, FES, FGF1, FMN1, FXYD1, LTBR, PHLDB1, SMAD3, SP100, STARD13, TAGLN. **c** Illustration of the process steps followed in the analysis of the cell samples presented in the study. Briefly, primary pBT cell cultures can be cultured in vitro under different conditions: 2D or 3D, in presence or absence of serum in culture media and at early or late passages. Analysis was conducted using Illumina BeadChip technology, enabling the acquisition of dual information encompassing DNAm and CNV profiles. The samples underwent classification utilizing the Brain Tumor Classifier and visualized in different clusters using t-SNE visualization. Based on methylation scores and CNV profiles, samples were systematically categorized into six different conditions, providing researchers with valuable tools to assess the faithfulness of their cell culture models before embarking on in vitro experiments. Created with BioRender.com.

Finally, we used these lists of hypo-/hyper-methylated genes to investigate whether there were shared active biological processes among the 4 unfaithful tumor-derived cell types according to t-SNE clustering (Fig. 6): we identified 22 shared genes (Fig. 6a), all hypomethylated, which were tested for GO enrichment. This analysis resulted in the identification of 10 biological processes whose activation is likely associated with altered expression patterns of cultured cells compared to the tumor of origin. Of note, this final set of hypomethylated genes are shared among unfaithful cells converging on response to growth factors and migration pathways: (i) signaling cascade activation (e.g., “zymogen activation” and “transmembrane receptor protein serine/threonine kinase signaling pathway”), (ii) tissue organization pathways (e.g., “mesenchyme development”, “angiogenesis” and “morphogenesis of an epithelium”), and (iii) cellular migration (“ameboidal-type migration”) (Fig. 6b).

## Discussion

Tumor research extensively uses model systems to investigate processes contributing to oncogenesis and disease progression as well as to identify potential therapeutic pathways in the preclinical setting. However, in many cases, the difficulty in culturing primary cells that retain the primary tumors’ genetic and epigenetic features, as well as the sparse number of available samples, could limit the implementation of pBT in vitro model systems^31^. Hence, there is an urgent need for developing models that faithfully recapitulate pathophysiology.

Recently, researchers have proposed various methods to determine the similarity of cancer models to natural tumors using genomic, transcriptomic, epigenomic, or ensemble molecular profiles^32–35^. Here we evaluated different cell culture parameters and developed a pipeline to assess the fidelity of our models using molecular traits, such as CNV and DNAm (Fig. 6c). Moreover, we described the functional relevance of these molecular profiles, highlighting common pathways involved in growth and differentiation of cultured cell lines. Our findings showed that cell lines cultured under stem cell enriching conditions (serum-free, 3D) and maintained in culture early passages are characterized by a higher degree of faithfulness, both in terms of DNAm and CNV profile, with respect to the counterparts maintained in 2D cultures, presence of serum, and for longer passages.

It is increasingly clear that 2D cultures do not mirror many primary tumor types, favoring clonal selection and genetic mutations, and that this effect is emphasized after multiple passages in culture, suggesting that long-term cultures can trigger processes leading cells to rearrange their DNAm status. To address this issue, 3D cultures, such as brain tumor spheroids and organoids, have recently been adopted thanks to the capability of this model to better mimic the physical and biochemical features of a solid tumor mass, also in pBT^36^. Several studies have shown that 3D culturing provides a more physiological environment compared to 2D cultures, allowing proper cell-cell and cell-ECM interactions^37^. Consistently, all unfaithful tumor cells (*i.e*., MB, HGG, LGG, and DMG) in our study had a similar epithelial-mesenchymal transition pattern.

The concept of monolayer/2D culture is closely related to time in culture and the number of passages; in fact, long passages cultures may induce, as an adaptive mechanism, a selection process of cells that allows the loss of certain tumor subpopulations and clonal expansions. An example is 3D models from glioblastoma that are genetically more representative of the primary tumor cells compared to 2D cultures (both cultured in serum medium), and that are more stable over time. On the other hand, molecular profiles of cells grown in 2D cells have changed considerably and progressively over time^38^.

Furthermore, the serum is commonly used in traditional 2D culture as a source of growth factors and nutrients, and in many cases, it is advantageous to cell growth even if some studies have demonstrated that it modulates proteins and gene expression^38,39^. In our experience, the presence of serum is not advantageous for cell growth as tumor-spheres because it induces differentiation, slowing down cell proliferation. Indeed, the presence of serum in the culture medium is another variable that is closely related to the fidelity of cellular models. Serum supplementation in cell cultures produces biological adverse effects on the global gene expression^40^ likely depending on an aberrant DNAm pattern. In line with these observations, our data suggest that serum significantly affects cell epigenetic features and reveals that the signaling cascade activation linked to the presence of growth factor in serum media is one of the main biological processes enriched in the unfaithful cell models.

Notably, LGG cell cultures exhibited the highest divergence (14%) and the most consistent cells at early passages (2/3). The distinctive behavior observed in LGG cell cultures can be attributed to specific characteristics of their proliferative capacity. Primary LGG cells exhibit a notably low proliferative rate, limited to a finite number of divisions in culture, after which the cells enter a phase of growth arrest and initiate replicative senescence. This senescence program is triggered by an aberrant activation of the MAPK pathway, a hallmark feature of these tumors, and of the p16 pathway^41^. Hence, late-passage LGG cells may not faithfully represent the authentic tumor cells that undergo senescence. Instead, these cells likely constitute a population of supportive cells sustained in culture due to the presence of serum in the media. Coherently, 94% of our late-passage LGG cell cultures result unfaithful. This consideration is crucial in interpreting our experimental data and underscores the importance of considering the specific cell culture conditions. On the other hand, EPN, MB, and DMG cell cultures were 100%, 71%, and 67% consistent with primary tumors, respectively. All the EPN and DMG cell cultures tested were maintained in serum-free conditions, like the faithful MB cell lines which were grown predominantly in serum-free and 3D conditions. Our results suggest that “early”, 3D, serum-free cells are the most reliable, and mainly that the presence of serum is the variant that discriminates consistent cells *vs* unfaithful ones in a significant way. Moreover, we observed that the “different” cells had a lower overall number of CNVs and a flatter profile than the tumors of the same histological type. We explain the observed difference in CNV patterns between tumors and tumor-derived cell cultures in part due to tumor heterogeneity. Tumors are composed of different cell types at distinct stages of differentiation. Cell cultures could select only a subset of clones/cell types, resulting in a more homogeneous cell population. Of note, it should be considered that the biopsy fragment used to generate the cell cultures is necessarily different from the one used for diagnosis. This issue is particularly critical for highly heterogeneous tumors, such as DMG^42^. Notably, in our study, DMG primary cells retained the faithful MC, most likely because histone mutation was an early event in tumorigenesis shared by all primary tumor cells^43^. However, in line with the biological heterogeneity of this tumor type (biopsy or culturing selection of individual subclone), two DMG cell lines derived from tissue T1 showed a striking loss of genetic alterations.

In conclusion, culture media, dimensionality, and passage number affect DNAm and CNV patterns. By comparing genes involved in the changed genome-wide DNAm patterns, we appreciated a significant enrichment of cellular processes and pathways correlating with the culturing method, highlighting the need to optimize culture conditions to maintain a coherent methylation profile in vitro. We strongly recommend serum-free and 3D cultures as they more frequently preserve the biological and molecular characteristics of the tumor of origin. Our study demonstrates that DNAm could be a useful tool for testing quality control of cell culture techniques and strategies, especially when paired with other methods such as DNA sequencing (for clonality detection) or bulk- or single-cell-RNA sequencing (to highlight changes in cellular heterogeneity). In conclusion, we advise its use when undertaking in vitro pBT studies.

## Methods

### Samples

In this study, a total of 241 genome-wide DNAm profiles were analyzed (Table 1 and Supplementary Table 1). We evaluated 155 different pediatric brain tumor tissues (T), clinically and histologically confirmed, for which high-quality DNA was available. Thirty-four samples of the entire tissues’ cohort were studied together with their derived cell lines (one or more cell lines for each tumor), the remaining 121 tissues did not have corresponding cells. The tissue cohort included 61 medulloblastomas (MB) divided into the four principal molecular subgroups: 10 and 17 tumors with activation of the WNT and Sonic hedgehog (SHH) pathways, respectively; 13 tumors classified within the MB-Group 3 and 21 tumors belonging to the MB-Group 4. We included a total of 81 glial tumors with several histotypes and 9 ependymomas (EPN). For easier execution of analyzes and interpretation of results, taking into consideration the type and numbers of the available primary cell lines, we decided to subdivide the glial tumors into low-grade gliomas (LGG, *n* = 45), and pediatric-type diffuse high-grade gliomas (*n* = 36). We further subdivided the latter into diffuse (pontine) midline gliomas H3 K27M-altered (DMG) (*n* = 13) and all the remaining high-grade gliomas subtypes (HGG) (*n* = 23). Lastly, we included 4 meningiomas (MNG) as a control group. The specific nomenclature for each tumor, according to 2021 WHO classification of Tumors of the Central Nervous System^44^ is reported in Table 1 and S1. The cell cohort included a total of 86 samples: 5 commercial cell lines, 9 organoids, 56 primary cells paired with corresponding tissues and 16 primary cell cultures without tumors (see Supplementary Table 1 and Fig. 1). Cell cultures were named according to the nomenclature below: primary cultures (C) in both two-dimensional (2D) and three-dimensional (3D) conditions, either as serum-free (SF) or serum-supplemented medium (S) and considering early (E) and long (L) passages in culture. Tumor tissues and cell lines are named with the tumor acronym and progressive numbers. The experimental workflow is outlined in Fig. 1.

### Cell cultures

pBT samples used for cellular models’ generation were obtained from patients operated at the Bambino Gesù Children’s Hospital under approvals by the Ethical Committee of Bambino Gesù Children’s Hospital [Protocol n° 1680_OPBG_2018 (28.12.2018); Protocol n° 1863_OPBG_2019, (01.08.2019); Protocol n° 3024_OPBG_2023 (01.02.2023); Protocol n° 21LB, Study Number 730/2013]. All samples were collected with written informed consent from the patients’ parents or legal guardians and in compliance with all relevant ethical regulations, including the Declaration of Helsinki.

Patient-derived MB/HGG stem-like cells (SLC) were obtained from primary human MBs and maintained in stem-cell medium (SCM), as previously described^45^: DMEM/F12 (Gibco) supplemented with 0.6% glucose, 25 mg/ml insulin, 60 mg/ml *N*-acetyl-l-cysteine, 2 mg/ml heparin, 20 ng/ml EGF, 20 ng/ml bFGF (Peprotech), 1% penicillin-streptomycin and B27 supplement without vitamin A (Gibco).

Patient-derived LGG cells were isolated and maintained in culture as previously reported^46,47^. Briefly, LGG cell lines were cultured in 2D in culture “*Method a*”: normal human astrocytes (NHA) complete medium (Lonza): ABMTM basal medium (CC-3187) supplemented with growth factors, cytokines); or culture “Method b”: samples were mechanically processed, washed twice with phosphate-buffered saline (PBS) and re-suspended in a media containing DMEM (Thermo Scientific) supplemented with 10% fetal bovine serum (Thermo Scientific), L-glutamine (EuroClone), penicillin/streptomycin (EuroClone), sodium pyruvate 100 mM (EuroClone)^48,49^.

MB cell lines (DAOY, D283, D341, and CHLA-01-Med) were obtained from the ATCC^50^. The lines were grown in Eagle’s Minimum Essential Medium (EuroClone) supplemented with 10% (DAOY) or 20% (D283 and D341) heat-inactivated fetal bovine serum (FBS, Gibco), 1% sodium pyruvate, 1% NEAA, 1% l-glutamine, and 1% penicillin-streptomycin. CHLA-01-Med cells were cultured in Dulbecco’s modified eagle medium: nutrient mixture F-12 (DMEM/F-12, Gibco) medium with B-27 supplement (Gibco), with 20 ng/ml EGF, 20 ng/ml bFGF (Peprotech), in addition to 1% L-glutamine and 1% penicillin-streptomycin.

KNS42 cells, established human HGG cell lines^51^ were purchased from the Japanese Collection of Research Bioresources Cell Bank. Cells were maintained in DMEM/F12 medium supplemented with 10% heat-inactivated fetal bovine serum (FBS, Gibco), 1% L-glutamine, and 1% penicillin-streptomycin^52^.

Patient-derived HGG and DMG cell lines were established and maintained in culture as previously described^42,53^. Briefly, they were cultured in a serum-free medium “Tumor Stem Media (TSM)”: Neurobasal (-A) (Invitrogen), and DMEM:F12 (Life Technologies) in a 1:1 ratio, supplemented with HEPES, NEAA, Glutamaxx, sodium pyruvate, and B27 supplement without vitamin A (Invitrogen), 20 ng/ml human-EGF, 20 ng/ml human bFGF, 10 ng/mL human PDGF-AA and 10 ng/mL PDGF-BB (Shenandoah) and 2 ng/mL heparin (Stem Cell Technologies). Cells were also grown in TSM under 2D adherent cultures on laminin (“*Method c*”).

Medulloblastoma organoids were derived from human iPSC cells as described^9^ and cultured as previously reported^54,55^.

We considered the threshold of 15 days/ 5 passages (p5) to define “late” LGG and MB primary cell lines. Indeed, LGG cells showed a fast-growing phase during the first 15 days (generally corresponding with p5) before slowing down and activating a senescent phenotype^46,47^. Similar data were observed in MB cell cultures (unpublished data).

For HGG and DMG, we considered “late” cells that have been in culture for more than 30 days and have passed passage 10.

**Fig. 7:**
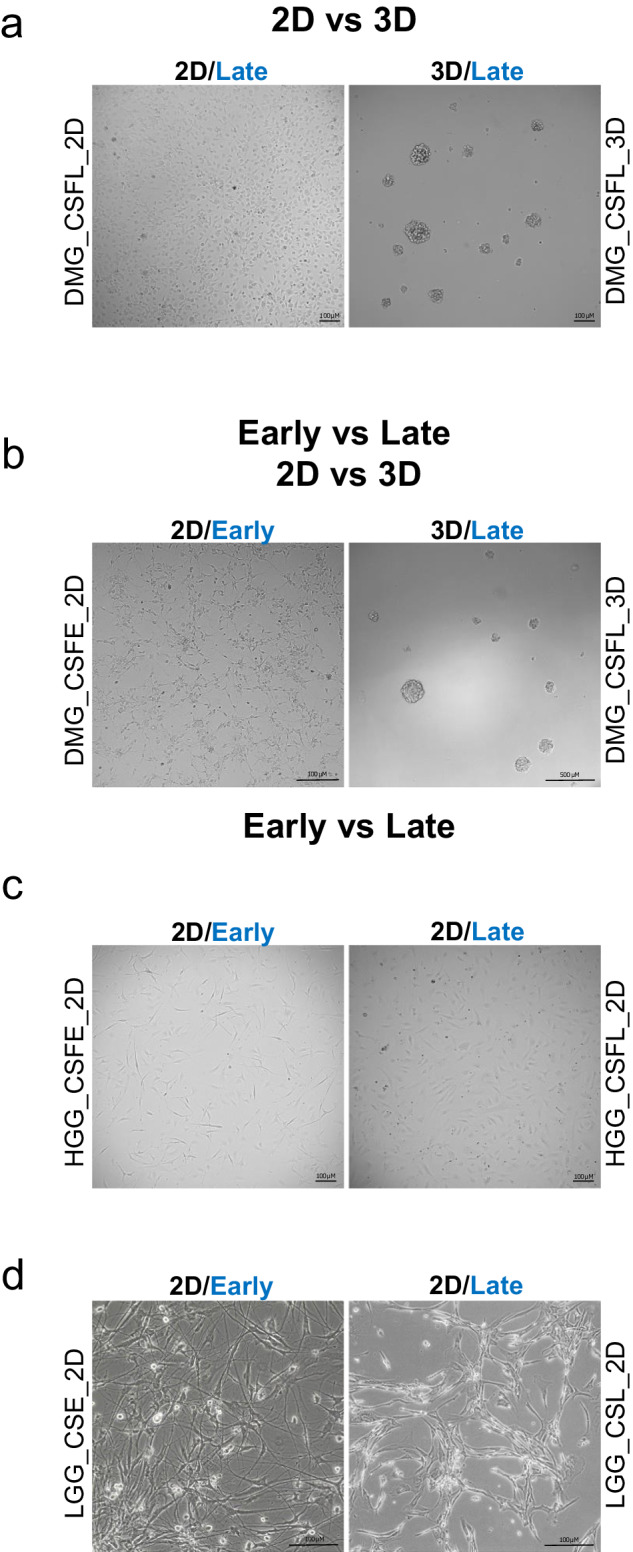
Representative brightfield images of primary patient-derived cell lines. **a** The DMG primary cells were cultured adherent on 2D (laminin) (left panel; scale bar 100 µM) or in 3D like neurospheres (right panel; scale bar 100 µM); both at late passages (p25). **b** DMG primary cells were cultured adherent on 2D (laminin) (left panel; scale bar 100 µM) or in 3D like neurospheres (right panel; scale bar 500 µm) in stem cell culture condition. The 2D cells are at early passages (p5) while the 3D cells are at late ones (p20). **c** The HGG primary cells were cultured adherent on 2D (laminin) at two different time points (p2 vs p13) (scale bar 100 µM). **d** The LGG primary cells were cultured adherent on 2D (in serum medium) at two different time points (p2 vs p15) (scale bar 100 µM). Images were acquired at Leica DMi8 at ×10 magnification and at Leica DM2500 microscope at ×20 magnification.

All analyzed cell line samples were derived from growing and healthy cultures (Fig. 7), and were mycoplasma-free and maintained in a humidified atmosphere containing 5% CO_2_ at 37 °C.

### DNA extraction

MagPurix extraction kit ZP02009 (Zinexts, Life Science Corporation) with Zinexts automated system was used for DNA extraction from formalin-fixed paraffin-embedded (FFPE) tissue specimens. Tumor areas with the highest tumor cell content (≥70%) were selected for DNA extraction. DNA extraction from organoids was previously described^9^; MagPurix extraction kit ZP02005 (Zinexts, Life Science Corporation) with Zinexts automated DNA extraction was used for cell cultures.

### DNA methylation profiling

DNAm profiling was performed after signed written consent was obtained from the patients’ parents or legal guardians in accordance with the Bambino Gesù Children’s Hospital Ethical Committee (Protocol n° 1556-OPBG, 01/15/2019), in compliance with all relevant ethical regulations including the Declaration of Helsinki. Samples were analyzed on Illumina iScan microarray platform using Illumina Infinium Human Methylation EPIC BeadChip arrays in accordance with the manufacturer’s protocols as previously described^56^. Briefly, 500 ng or 250 ng of DNA were used as input material for cell lines and FFPE tissues, respectively. DNA quantification was performed with the Qubit® dsDNA BR Assay Kit (Thermo Fisher Scientific). Bisulfite conversion was facilitated with the Zymo EZ Methylation Kit (Zymo Research Irvine) followed by purification with Zymo DNA Clean Kit (Zymo Research Irvine). DNA from FFPE tissue was treated with the Infinium HD FFPE Restore Kit prior to hybridization to the Infinium BeadChip (Illumina). To minimize systematic bias, the samples were randomly distributed onto the BeadChips, which hold eight samples per array. The sample methylation data were categorized using the brain tumor classifier v12.5 (, accessed on 06/01/2023)^57^, which also generated the copy number variation (CNV) plots. Data analysis was performed as previously described^9,13^. IDAT files were imported into R V.4.0.5 to be analyzed with fit-for-purpose packages. A series of probe-filtering steps and checks for a batch effect were performed to assess data quality. To this aim, probes with detection *p*-value > 0.01 and those with less than 3 beads in at least 5% of samples were removed. Afterward, probes containing SNPs at or near the CpG sites, located on X/Y chromosomes and those known to cross-react with multiple genome locations, according to Nordlund et al.^58^. were excluded. The presence of batch effect and/or outliers was assessed by principal component analysis (PCA) performed over all filtered probes as well as by checking the similarity of samples based on the top 1000 most variable probes by Multidimensional scaling (MDS). Beta values were then normalized using the Beta Mixture Quantile dilation (BMIQ) R package^59^. A further check for any sample deviating from the characteristic beta value bimodal distribution was performed to confirm the absence of outlier samples. Singular value decomposition (SVD) analysis^60^ was carried out to estimate both technical and biological sources of variation in our experiments. The *champ.SVD()* function^61^ with default parameters was used for this analysis, setting covariates as follows: Sample group (Tumors/Cells/Organoids), Sex, Age, Tumor Classification (DMG/EPN/HGG/LGG/MB/MNG), Sample material (FFPE/frozen), Time of culture (early/late), Slide, Slide position.

Unsupervised dimensionality reduction was applied to the whole normalized methylation dataset to explore relations among the different tumors and cell types. The original structure of data was not perturbed, considering that the relatively small size of our cohort allowed handling data without the use of any feature selection technique^62^. Referring to the t-SNE protocol by Kobak and Berens^63^ 20-component PCA initialization was performed, followed by multiple t-SNE to determine which value of hyper-parameters resulted in the best cluster aggregation/separation. We varied perplexity ranging from 5 to 100, theta from 0.1 to 1, and early exaggeration iterations (e.e.i.) from 100 to 500. A thorough visual comparison of results led us to set as final hyper-parameters theta=0.5, e.e.i.=200, perplexity=15. The snifter v1.4.0 R wrapper (https://rdrr.io/bioc/snifter/) of the python openTSNE library for the fast interpolation-based t-SNE was used to perform the analysis^62,64^.

t-SNE cluster definition and stability were assessed by hierarchical density-based spatial clustering of applications with noise (HDBSCAN), available in the dbscan R package v1.18^65,66^. We set the minimum cluster size parameter to four, equal to the number of samples that make up the minor group of tumor tissue type which is meningioma. Cluster stability was evaluated using the density-based clustering validation (DBCV) metric^67^.

Differentially methylated regions (DMR) analysis took advantage of DMRcate v2.8.1R package^68^ using log_2_ transformed beta-values (M-values), FDR < 0.05, the gap between significant probes set to 1000 bp, and 7 as the minimum number of CpGs per region. For each sample of the test group, we calculated the difference between the beta values of that sample and the average beta values over samples of the reference group. By making averages both over the probes within the DMR and samples of the test group, we could therefore assign each DMR a unique value (Δβ) which reflects methylation difference. These analyses were performed only over DMRs localized in promoter regions and/or first exon (TSS200, TSS1500, 1stExon). We considered >10% of |Δβ| as the threshold for hypo- and hyper-methylated DMRs. The missMethyl R package v1.28.0 allowed us to assess the identified DMRs for significant gene and pathway enrichments using all CpGs as background, taking advantage of Wallenius’ noncentral hypergeometric test^68^. Gene Ontology (GO) and KEGG pathway enrichment were tested with the *GOregion* function of missMethyl, whereas the *gsaregion* function was used to perform GSA with Hallmark gene set testing provided by the Molecular Signatures Database (MSigDB) v7.1. Furthermore, the lists of significant genes were extracted from the missMethyl output to apply two additional methods for pathway enrichment discovery by means of Fisher’s exact test, that is, the clusterProfiler v4.0^69^ (*enrichGO* function, using ont=BP and qvalueCutoff = 0.05 parameters) and WebGestalt 2019 (ORA analysis, functional db=GO) tool^70^. The redundancy of enriched GO terms was reduced by means of the *simplify* function with a similarity cutoff 0.3/0.4, depending on the number of terms.

### CNV analysis by DNA methylation arrays

We performed CNV analysis on DNAm array data of primary cell lines with paired corresponding tumor tissues, using R package Conumee (https://bioconductor.org/packages/devel/bioc/vignettes/conumee/inst/doc/conumee.html)^57^.

First, we normalized the combined intensity values of both ‘methylated’ and ‘unmethylated’ probes comparing them to a set of copy-neutral controls, processed with the same array type as our samples, obtained through package CopyNeutralIMA (https://bioconductor.org/packages/CopyNeutralIMA). Then, we combined the intensities of neighboring probes resulting in 25,752 “bins” (regions with a homogenous signal intensity, a minimum size of 50 Kb and a minimum of 15 probes) per sample. To evaluate CNV similarity, we calculated the correlation value between the bins of each cell line and its paired tissue. According to the correlation values to their tissue of origin, we defined samples in the lower tertile as “different”, in the second tertile as “similar”, and in the top tertile as “coincident”. We also used conumee to generate whole-genome CNV plots to facilitate visual inspection of the results and detect segmental alterations. To examine how cell lines with “different” profiles diverged from tumor tissues of the same histology, we compared the mean number of regions where the absolute log2R value was higher than 0.1, and generated cumulative genome-wide CNV plots for sample groups of interest with package GenVisR^71^.

### Reporting summary

Further information on research design is available in the Nature Research Reporting Summary linked to this article.

### Supplementary information

REPORTING SUMMARY

Supplementary Info

Supplementary Table

Supplementary Table

Supplementary Table

Supplementary Table

## Data Availability

Genome-wide DNAm array raw data have been deposited in NCBI’s Gene Expression Omnibus (GEO - GEO Series accession number GSE225810) and are accessible through https://www.ncbi.nlm.nih.gov/geo/query/acc.cgi?acc=GSE225810.

## Code availability

The software used in this study is publicly available with software packages, versions and parameters described in detail in the Methods section.
