# Supplementary material for: Evaluating cell culture reliability in pediatric brain tumor primary cells through DNA methylation profiling
Source: NPJ Precis Oncol. 2024 Apr 18;8:92. doi: 10.1038/s41698-024-00578-x (PMC11026496; doi:10.1038/s41698-024-00578-x)
Supplement: Supplementary file 1 — REPORTING SUMMARY [file 41698_2024_578_MOESM1_ESM.pdf]

nature portfolio

Corresponding author(s): Andrea Ciolfi, Evelina Miele

Last updated by author(s): Feb 20, 2024

Reporting Summary

Nature Portfolio wishes to improve the reproducibility of the work that we publish. This form provides structure for consistency and transparency in reporting. For further information on Nature Portfolio policies, see our Editorial Policies and the Editorial Policy Checklist.

Statistics

For all statistical analyses, confirm that the following items are present in the figure legend, table legend, main text, or Methods section.

n/a	Confirmed	
☐	☒	The exact sample size (*n*) for each experimental group/condition, given as a discrete number and unit of measurement
☒	☐	A statement on whether measurements were taken from distinct samples or whether the same sample was measured repeatedly
☐	☒	The statistical test(s) used AND whether they are one- or two-sided<br>*Only common tests should be described solely by name; describe more complex techniques in the Methods section.*
☒	☐	A description of all covariates tested
☒	☐	A description of any assumptions or corrections, such as tests of normality and adjustment for multiple comparisons
☐	☒	A full description of the statistical parameters including central tendency (e.g. means) or other basic estimates (e.g. regression coefficient) AND variation (e.g. standard deviation) or associated estimates of uncertainty (e.g. confidence intervals)
☐	☒	For null hypothesis testing, the test statistic (e.g. F, t, r) with confidence intervals, effect sizes, degrees of freedom and P value noted<br>*Give P values as exact values whenever suitable.*
☒	☐	For Bayesian analysis, information on the choice of priors and Markov chain Monte Carlo settings
☐	☒	For hierarchical and complex designs, identification of the appropriate level for tests and full reporting of outcomes
☒	☐	Estimates of effect sizes (e.g. Cohen's *d*, Pearson's *r*), indicating how they were calculated

Our web collection on statistics for biologists contains articles on many of the points above.

Software and code

Policy information about availability of computer code

Data collection	Samples were analyzed on Illumina iScan microarray platform using Illumina Infinium Human Methylation EPIC BeadChip arrays in accordance with the manufacturer's protocols.
Data analysis	IDAT files were imported into R V.4.0.5. ChAMP v2.21 was used for QC and import of IDAT files. The snifter v1.4.0 R wrapper of the python openTSNE library was used for t-SNE analysis, and cluster definition and stability were assessed by hierarchical density-based spatial clustering of applications with noise (HDBSCAN), available in the dbscan R package v1.18. Differentially methylated regions (DMR) analysis took advantage of DMRcate v2.8.1 R package. MissMethyl R package v1.28.0 and ClusterProfiler v4.0 were used for genes set enrichment analyses and clustering.

For manuscripts utilizing custom algorithms or software that are central to the research but not yet described in published literature, software must be made available to editors and reviewers. We strongly encourage code deposition in a community repository (e.g. GitHub). See the Nature Portfolio guidelines for submitting code & software for further information.

Data

Policy information about availability of data

All manuscripts must include a data availability statement. This statement should provide the following information, where applicable:

- Accession codes, unique identifiers, or web links for publicly available datasets
- A description of any restrictions on data availability
- For clinical datasets or third party data, please ensure that the statement adheres to our policy

Genome-wide DNAm array raw data have been deposited in NCBI's Gene Expression Omnibus (GEO- GEO Series accession number GSE225810) and are accessible through https://www.ncbi.nlm.nih.gov/geo/query/acc.cgi?acc= GSE225810.

Research involving human participants, their data, or biological material

Policy information about studies with human participants or human data. See also policy information about sex, gender (identity/presentation), and sexual orientation and race, ethnicity and racism.

Reporting on sex and gender	Sex as biological attribute has been considered
Reporting on race, ethnicity, or other socially relevant groupings	NA
Population characteristics	NA
Recruitment	NA
Ethics oversight	DNAm profiling was performed after signed written consent was obtained from the patients' parents or legal guardians in accordance with protocols approved by the institutional review board (Protocol n° 1556-OPBG, 01/15/2019)

Note that full information on the approval of the study protocol must also be provided in the manuscript.

Field-specific reporting

Please select the one below that is the best fit for your research. If you are not sure, read the appropriate sections before making your selection.

☒ Life sciences ☐ Behavioural & social sciences ☐ Ecological, evolutionary & environmental sciences

For a reference copy of the document with all sections, see nature.com/documents/nr-reporting-summary-flat.pdf

Life sciences study design

All studies must disclose on these points even when the disclosure is negative.

Sample size	No Sample size calculation was performed due to the descriptive nature of this paper
Data exclusions	samples were excluded for low DNA quality
Replication	unique samples analyses
Randomization	no randomization was used due to the descriptive nature of this study
Blinding	blinding was not used due to the descriptive nature of this study

Reporting for specific materials, systems and methods

We require information from authors about some types of materials, experimental systems and methods used in many studies. Here, indicate whether each material, system or method listed is relevant to your study. If you are not sure if a list item applies to your research, read the appropriate section before selecting a response.

Materials & experimental systems

n/a	Involved in the study	
☒	☐	Antibodies
☐	☒	Eukaryotic cell lines
☒	☐	Palaeontology and archaeology
☒	☐	Animals and other organisms
☐	☒	Clinical data
☒	☐	Dual use research of concern
☒	☐	Plants

Methods

n/a	Involved in the study	
☒	☐	ChIP-seq
☒	☐	Flow cytometry
☒	☐	MRI-based neuroimaging

Eukaryotic cell lines

Policy information about cell lines and Sex and Gender in Research

Cell line source(s): ATCC and Japanese Collection of Research Bioresources Cell Bank

Authentication: Certificate from the referred cell bank

Mycoplasma contamination: All cells were tested for mycoplasma and resulted mycoplasma free

Commonly misidentified lines (See ICLAC register): NA

Clinical data

Policy information about clinical studies

All manuscripts should comply with the ICMJE guidelines for publication of clinical research and a completed CONSORT checklist must be included with all submissions.

Clinical trial registration: NA

Study protocol: NA

Data collection: NA

Outcomes: NA

Plants

Seed stocks: NA

Novel plant genotypes: NA

Authentication: NA
