# Supplementary material for: Evaluating cell culture reliability in pediatric brain tumor primary cells through DNA methylation profiling
Source: NPJ Precis Oncol. 2024 Apr 18;8:92. doi: 10.1038/s41698-024-00578-x (PMC11026496; doi:10.1038/s41698-024-00578-x)
Supplement: Supplementary file 2 — Supplementary Info [file 41698_2024_578_MOESM2_ESM.pdf]

Evaluating cell culture reliability in pediatric brain tumor primary cells through DNA methylation profiling

Supplementary Data

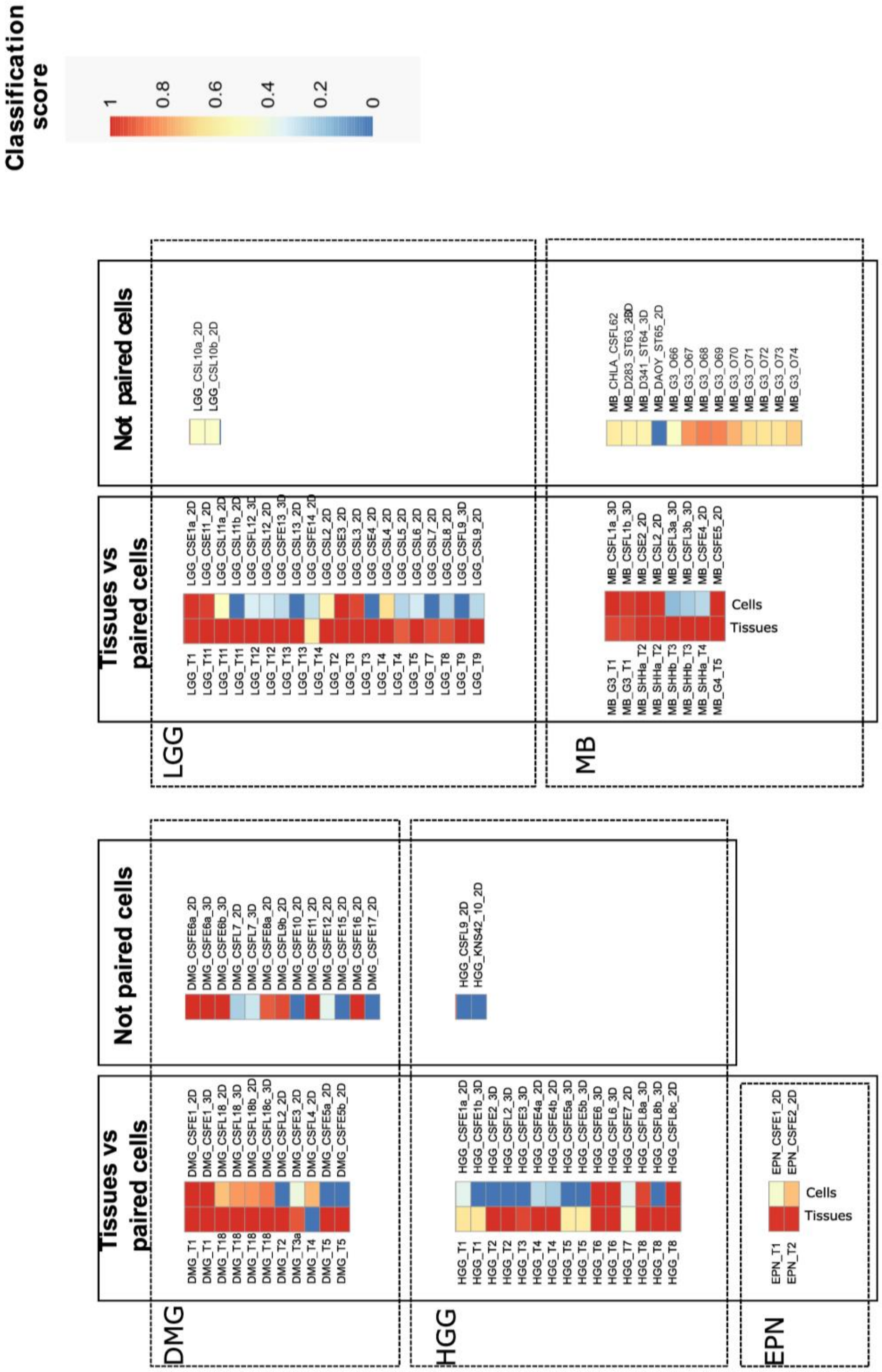

Supplementary Figure 1. Classification scores of all cell lines

Heatmaps representing the classification scores of 56 tumor tissues with their paired cell lines ("Tissues vs paired cells" panels) and the classification scores of 30 cell lines not paired with their tissue of origin ("Not paired cells" panels). The heatmaps are grouped by tumor type (DMG, HGG, EPN, LGG, MB) with dotted squares. Classification score goes from highest (red) to lowest (blue).

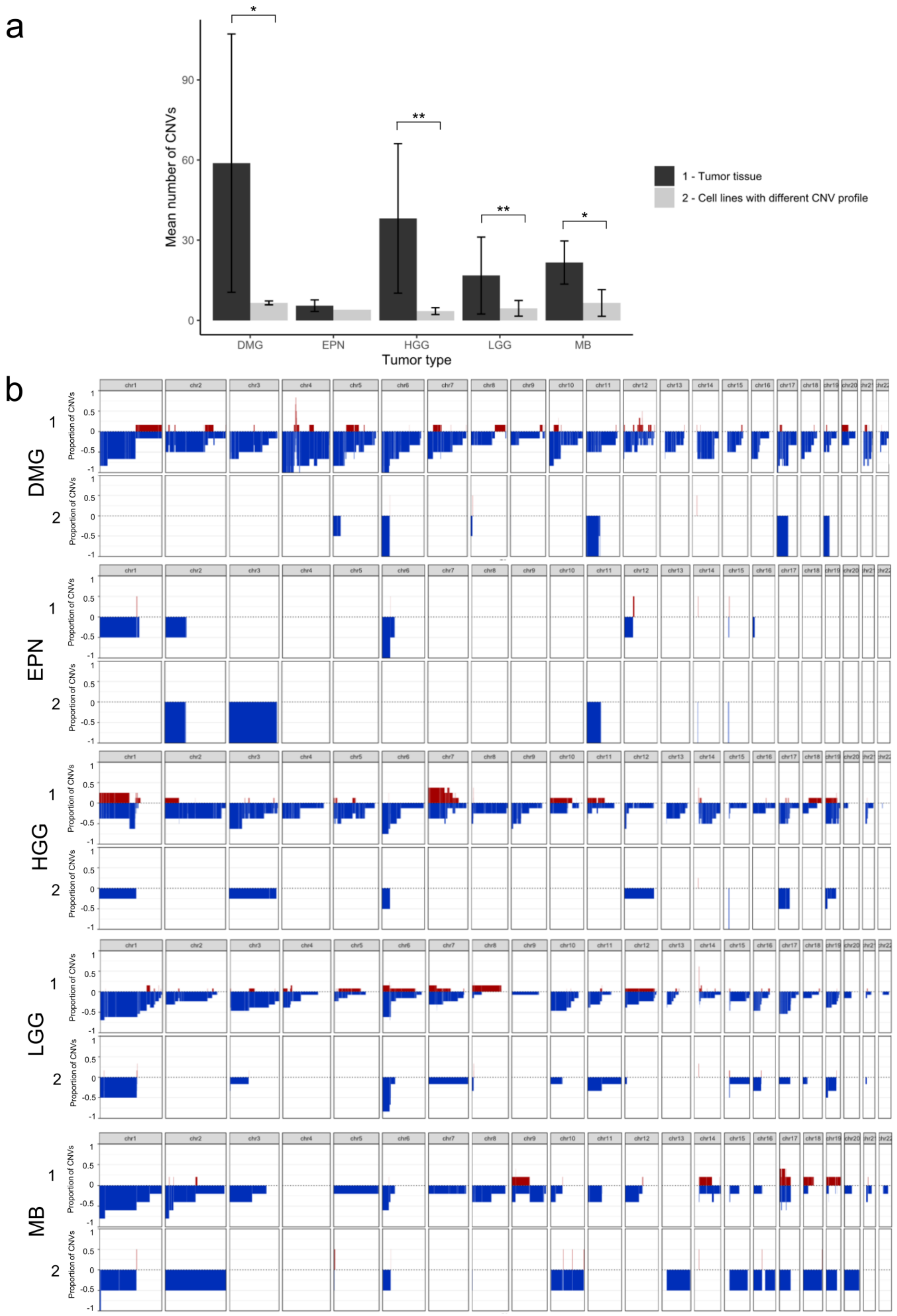

a
Mean number of CNVs
Tumor type
DMG
EPN
HGG
LGG
MB
1 - Tumor tissue
2 - Cell lines with different CNV profile
b
DMG
EPN
HGG
LGG
MB
Proportion of CNVs

Supplementary Figure 2. CNV analysis

A-Bar plot representing the number of CNVs with log2R>=0.1 or log2R<=-0.1 called with conumee package. For each histological tumor type, the mean number of CNVs and its standard deviation is represented for tumor tissues (dark grey) and cells of the same histology with «different» CNV profile. The brackets indicate the statistical significance of the differences between the sample types (*-p<0.05, **-p<0.001).

B-Cumulative CNV plots representing the proportion of copy number gains (log2R>=0.1, red) or losses (log2R<=-0.1, blue) across all chromosomes in tumor tissues (top) and cells of the same histology with different CNV profile (bottom) of each histological tumor type.

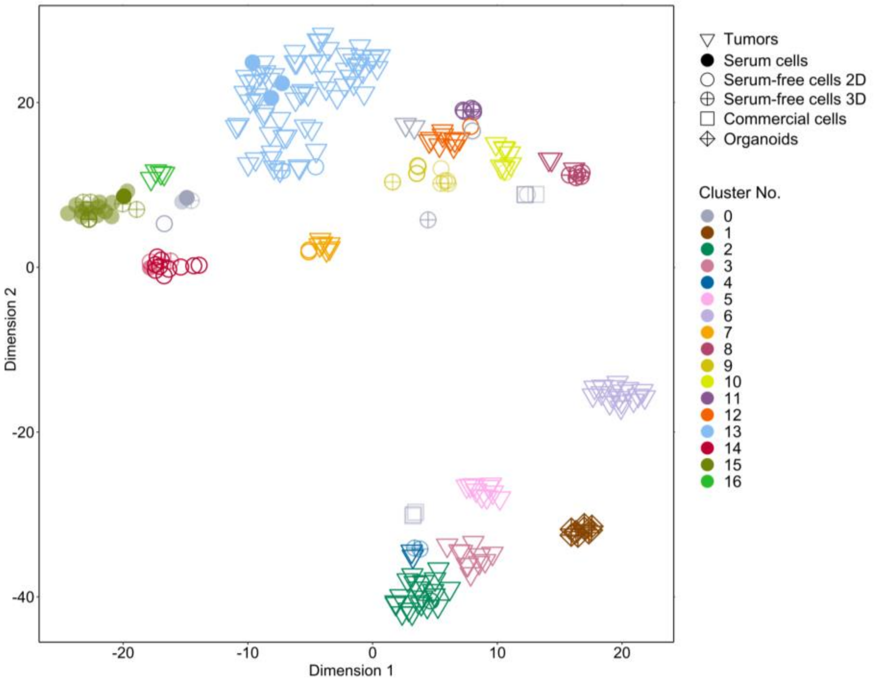

Supplementary Figure 3. Methylation data clustering by means of unsupervised learning.

Clusters identified over the t-SNE projection by applying the Hierarchical Density-Based Spatial Clustering (HDBSCAN) method. Gray color refers to outliers (noise points), other colors identify different stable clusters regardless of the tumor type. The shape of points indicates the type of sample (tumor, cells), culture media and cells culture method.

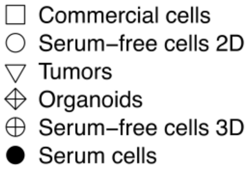

ID	sample	condition
**26**	**MB_G3_T1**	
432	MB_CSFL1a_3D	1
433	MB_CSFL1b_3D	1
**101**	**MB_SHHa_T2**	
439	MB_CSE2_2D	5
440	MB_CSL2_2D	6
**383**	**MB_SHHb_T3**	
382	MB_CSFL3a_3D	5
374	MB_CSFL3b_3D	5
**248**	**MB_SHHa_T4**	
706	MB_CSFE4_2D	6
**60**	**MB_G4_T5**	
707	MB_CSFE5_2D	1

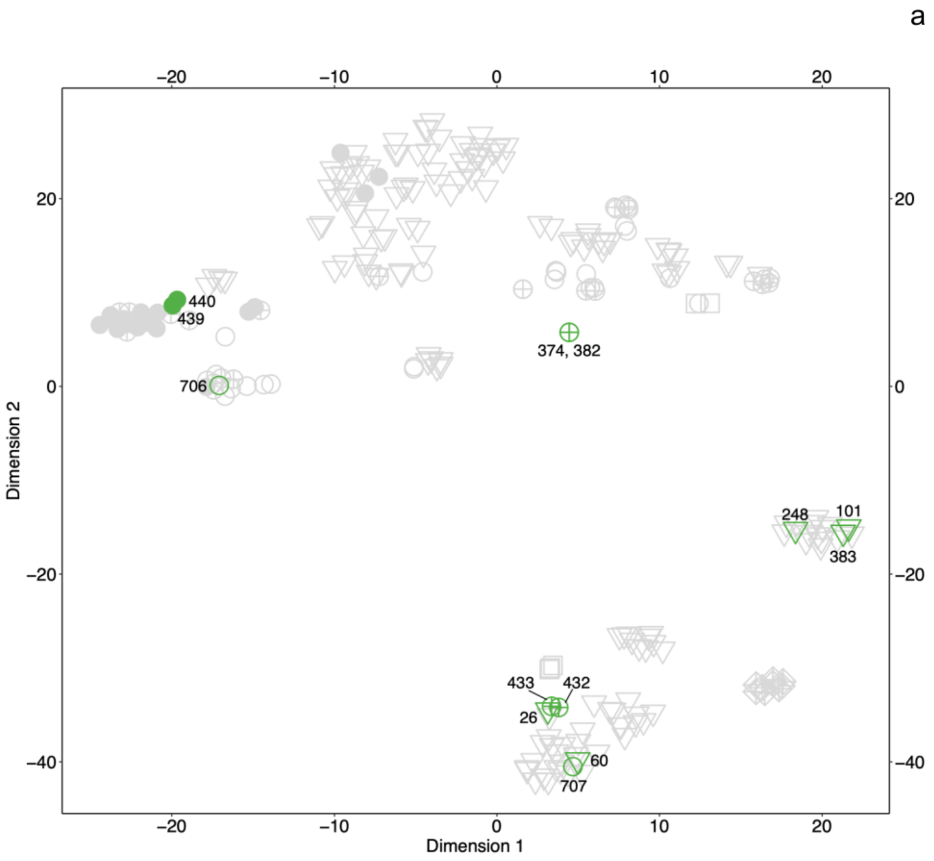

ID	sample	condition
**405**	**LGG_T1**	
409	LGG_CSE1a_2D	2
**406**	**LGG_T2**	
410	LGG_CSL2_2D	5
**407**	**LGG_T3**	
411	LGG_CSL3_2D	5
413	LGG_CSE3_2D	1
**418**	**LGG_T4**	
430	LGG_CSL4_2D	5
415	LGG_CSE4_2D	5
**419**	**LGG_T5**	
416	LGG_CSL5_2D	6
**446**	**LGG_T6**	
445	LGG_CSL6_2D	6
**183**	**LGG_T7**	
184	LGG_CSL7_2D	6
**448**	**LGG_T8**	
449	LGG_CSL8_2D	6
**252**	**LGG_T9**	
176	LGG_CSL9_2D	5
175	LGG_CSFL9_3D	6
**404**	**LGG_T11**	
412	LGG_CSE11_2D	2
408	LGG_CSL11a_2D	5
185	LGG_CSL11b_2D	6
**251**	**LGG_T12**	
177	LGG_CSL12_2D	5
178	LGG_CSFL12_3D	5
**337**	**LGG_T13**	
371	LGG_CSL13_2D	5
372	LGG_CSFE13_3D	5
**397**	**LGG_T14**	
912	LGG_CSFE14_2D	5

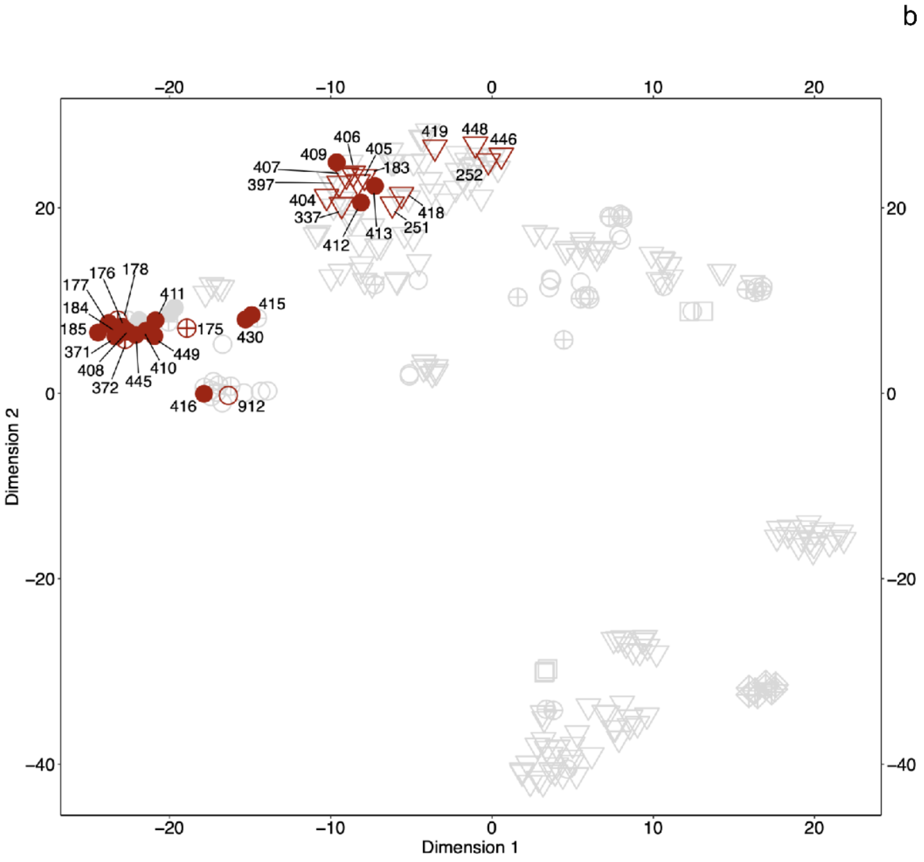

c

ID	sample	condition
**179**	**HGG_T1**	
180	HGG_CSFE1a_2D	1
181	HGG_CSFE1b_3D	4
**33**	**HGG_T2**	
182	HGG_CSFL2_3D	6
76	HGG_CSFE2_3D	6
**64**	**HGG_T3**	
74	HGG_CSFE3_3D	4
**62**	**HGG_T4**	
483	HGG_CSFE4a_2D	6
484	HGG_CSFE4b_2D	6
**441**	**HGG_T5**	
443	HGG_CSFE5a_3D	4
444	HGG_CSFE5b_3D	4
**417**	**HGG_T6**	
429	HGG_CSFL6_3D	1
414	HGG_CSFE6_3D	1
**140**	**HGG_T7**	
143	HGG_CSFE7_2D	1
**27**	**HGG_T8**	
427	HGG_CSFL8a_3D	5
428	HGG_CSFL8b_3D	5
162	HGG_CSFL8c_2D	1

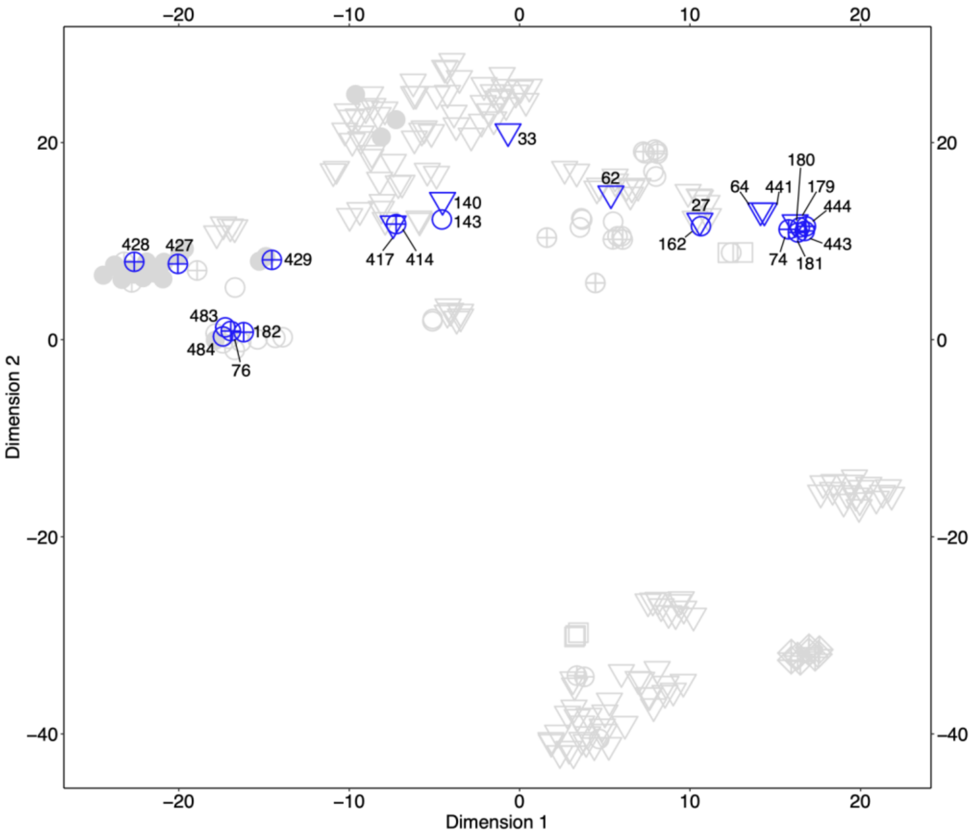

d

ID	sample	condition
**63**	**DMG_T1**	
451	DMG_CSFE1_2D	3
450	DMG_CSFE1_3D	3
**256**	**DMG_T2**	
79	DMG_CSFL2_2D	5
**249**	**DMG_T3a**	
**66**	**DMG_T3b**	
75	DMG_CSFE3_2D	1
**482**	**DMG_T4**	
394	DMG_CSFL4_2D	2
**456**	**DMG_T5**	
910	DMG_CSFE5a_2D	4
911	DMG_CSFE5b_2D	5
**130**	**DMG_T18**	
1095	DMG_CSFL18b_2D	1
1096	DMG_CSFL18c_3D	1
164	DMG_CSFL18_2D	1
165	DMG_CSFL18_3D	1

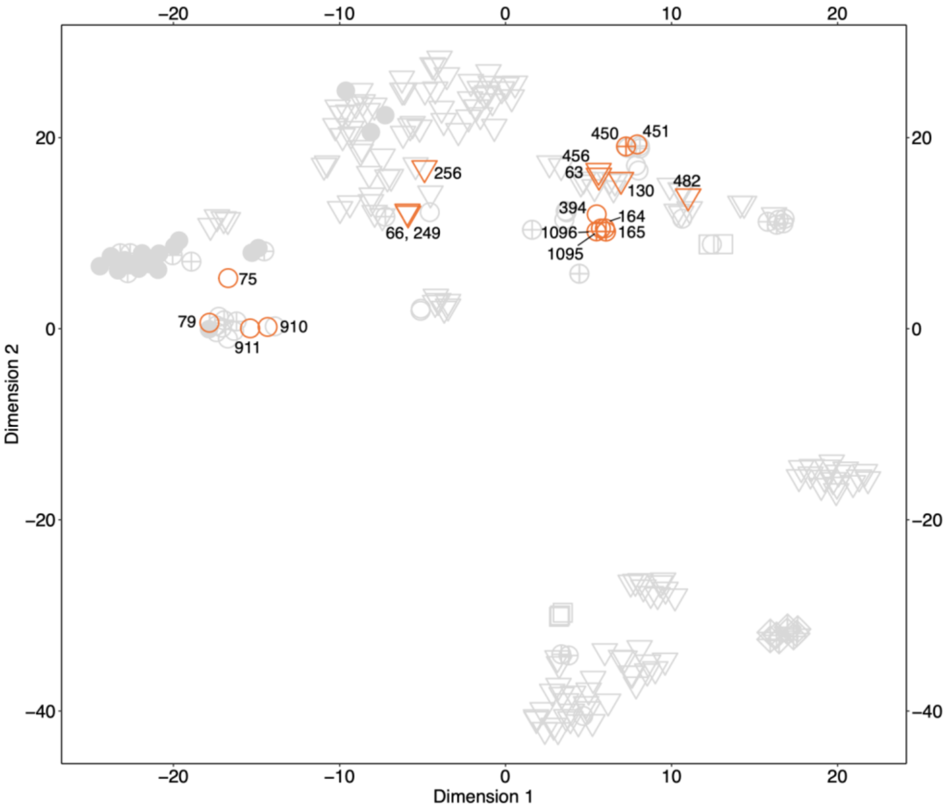

e

ID	sample	condition
**348**	**EPN_T1**	
611	EPN_CSFE1_2D	2
**521**	**EPN_T2**	
610	EPN_CSFE2_2D	3

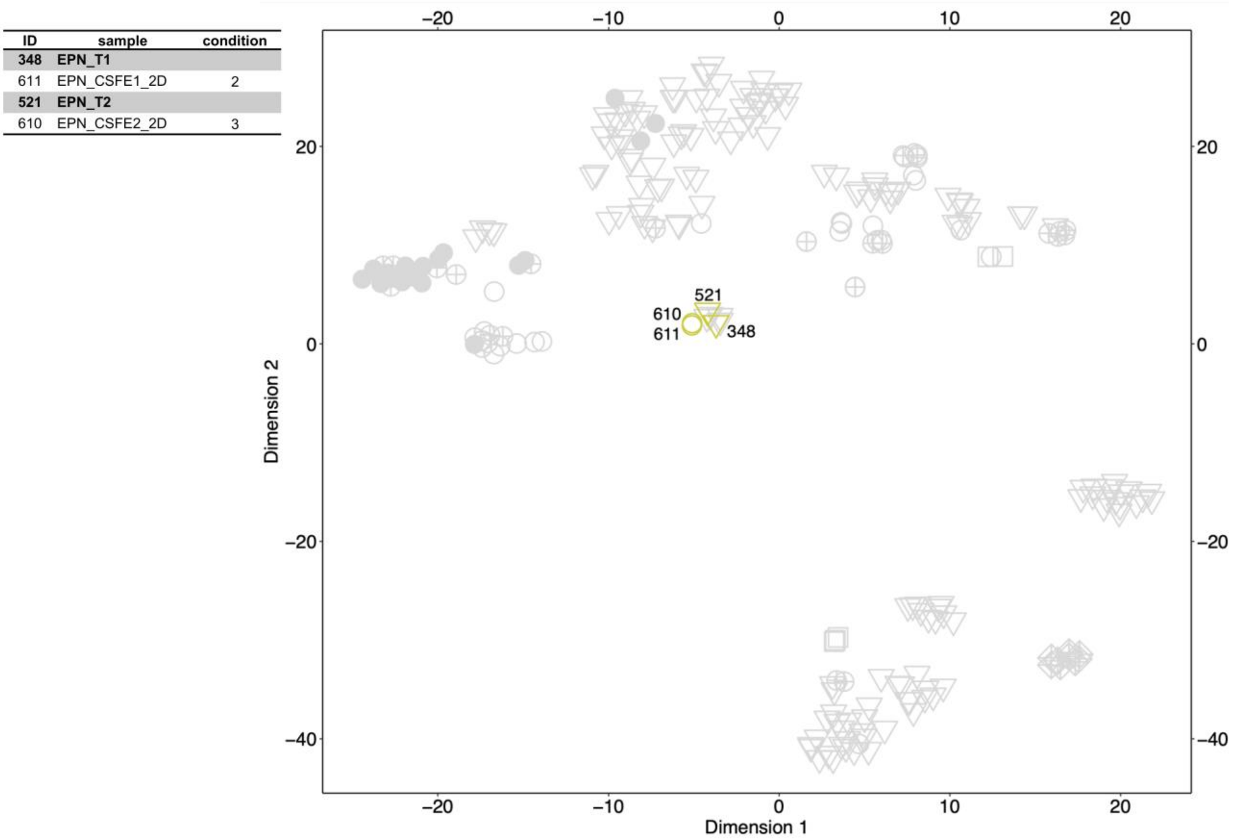

f

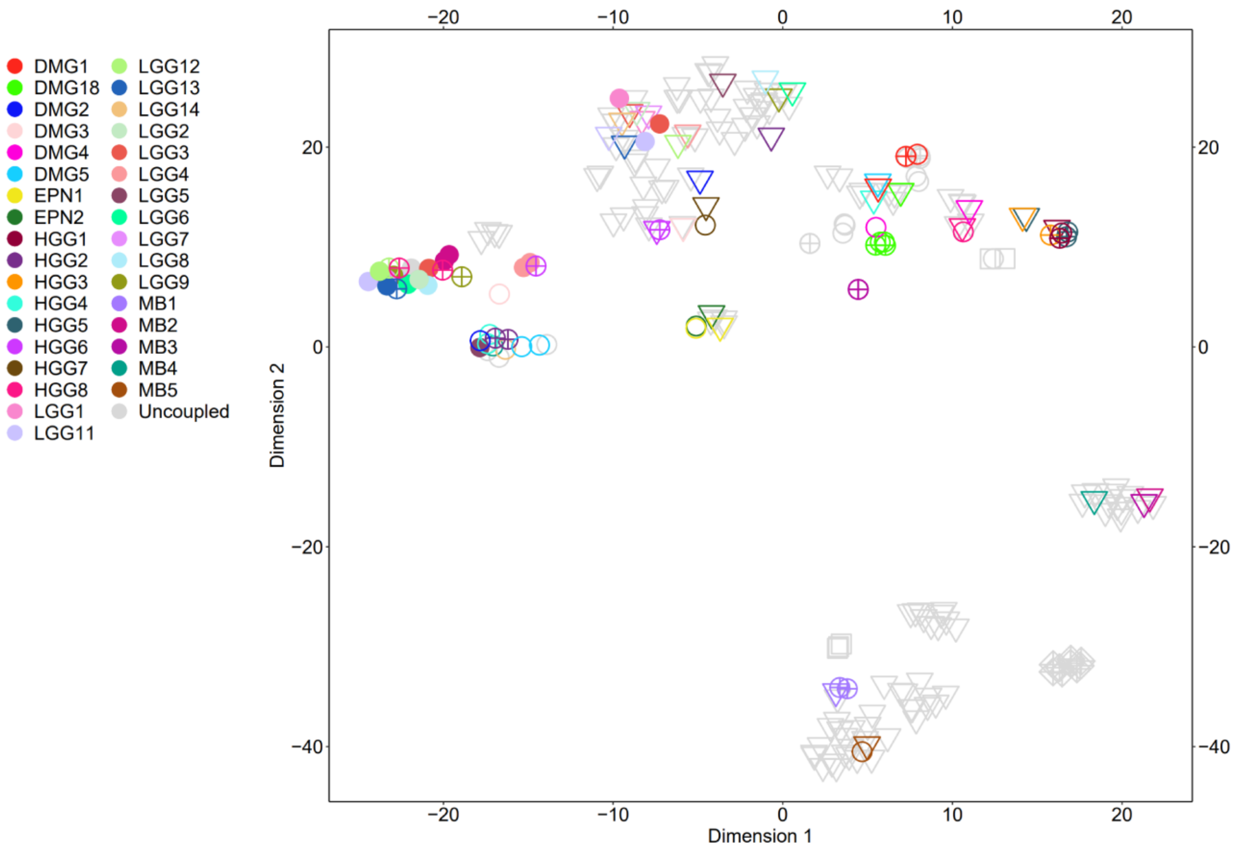

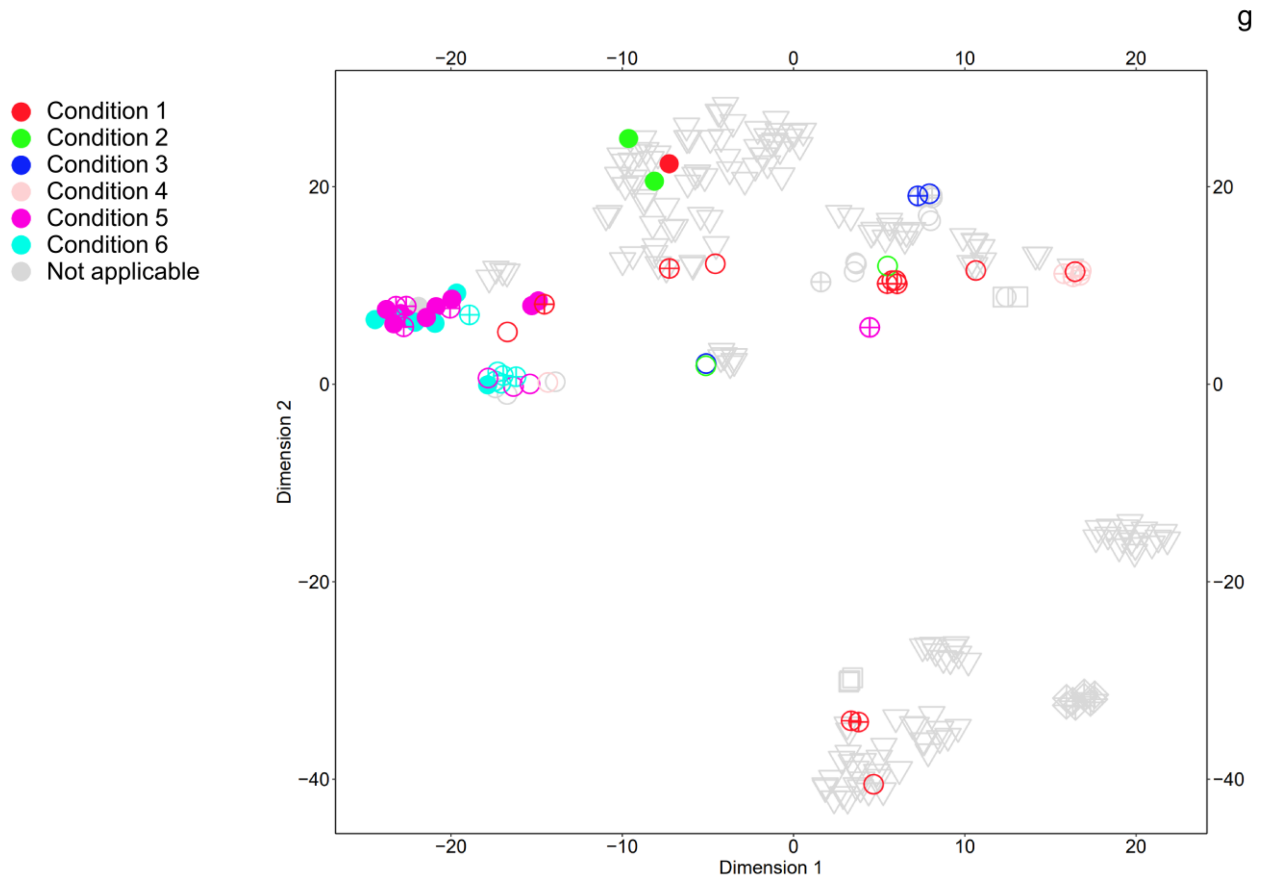

Supplementary Figure 4. Global structure 2D projection of methylation data detailed for paired samples and fidelity condition.

t-SNE analysis of DNAm data of 155 tissues and 86 tumor-derived cell lines as calculated in Figure 5 (A-E). On each panel, a different tumor type is highlighted with corresponding cell lines by means of a specific color and ID code MB (A), LGG (B), HGG (C), DMG (D), EPN (E). tSNE plot with different colors for each pair of tumor tissue and corresponding cell lines (F), and a tSNE plot with different colors for the 6 conditions (G).

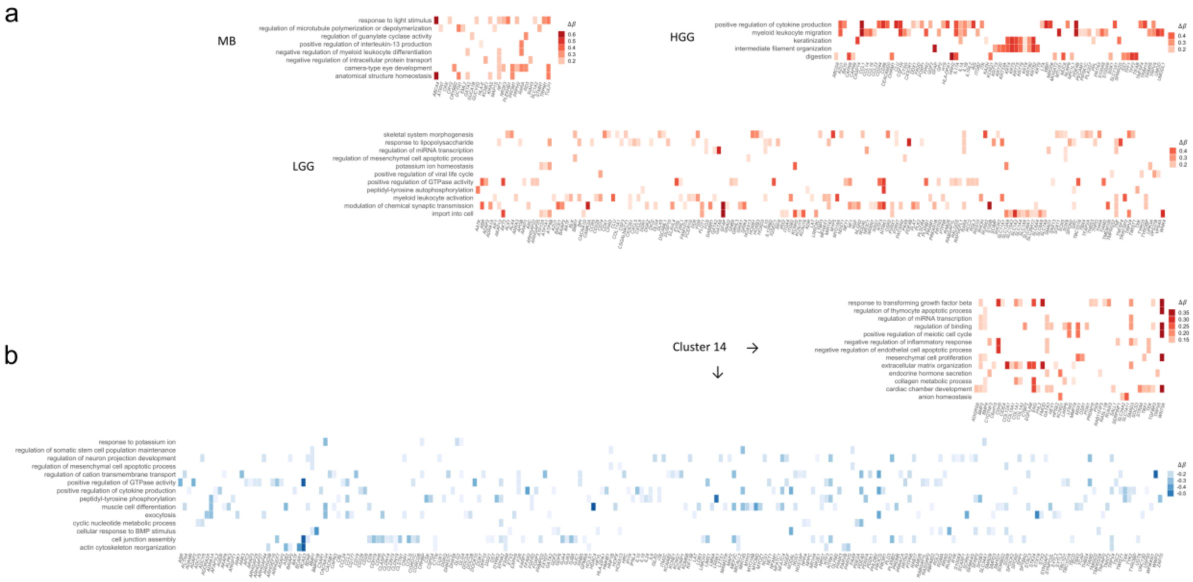

Supplementary Figure 5. Enriched Gene Ontology terms resulting from hyper- or hypomethylated genes.

Differential methylation patterns related to hypermethylated regions found in unfaithful cells of the LGG, HGG, MB cell lines (A). No significant enriched terms were found for unfaithful DMG cells. In parallel, heatmaps of the enrichment analysis over DMRs of cluster #14 compared to #15 are shown, for both the hypermethylated and hypomethylated status (B).

Supplementary Table 1. List of pBT cohort (tissue and cell cultures), histopathological data, methylation data and cell culture conditions.

Features of cell culture cohort and analysis of "faithfulness" based on methylation data. MC: Methylation class; L: long passages in culture; E: early passages in culture; SF: serum-free; S: serum-supplemented medium;2 D: two-dimensional culture; 3D: three-dimensional. Unfaithful: classifier score<0.3; useful: score between 0.3 and 0.84; faithful: score>0.84. Genome-wide DNAm array raw data have been deposited in NCBI's Gene Expression Omnibus (GEO- GEO Series accession number GSE225810) and are accessible through https://www.ncbi.nlm.nih.gov/geo/query/acc.cgi?acc= GSE225810.

Supplementary Table 2. Statistical analysis of cell score distribution for each cell cultures category.

Chi-square test was performed for each comparison. L: long passages in culture; E: early passages in culture; SF: serum-free; S: serum-supplemented medium;2 D: two-dimensional culture; 3D: three-dimensional. Unfaithful: classifier score<0.3; useful: score between 0.3 and 0.84; faithful: score> 0.84.

Supplementary Table 3. Description of CNV profile and identification of six different conditions combining methylation and CNV status.

L: long passages in culture; E: early passages in culture; SF: serum-free; S: serum-supplemented medium; 2D: two-dimensional culture; 3D: three-dimensional; Coincident: correlation>=0.55; Similar: 0.15<correlation<0.55; Different: correlation<0.55. MC: Methylation class.

Supplementary Table 4. Statistical analysis of cell CNV distribution for each cell cultures category and chi-square test performed for each comparison.

L: long passages in culture; E: early passages in culture; SF: serum-free; S: serum-supplemented medium; 2D: two-dimensional culture; 3D: three-dimensional.

Supplementary Table 5. Pediatric brain tumors (pBTs) and cell lines clustering results according to DNAm genome-wide profiling.

Cluster	0	1	2	3	4	5	6	7	8	9	10	11	12	13	14	15	16
Samples	MB_D341_ST64_3D	MB_G3_O74	MB_G4_T9	MB_G4_T14	MB_G3_T20	MB_WNT_T8	MB_SHHb_T61	EPN_T6	HGG_T5	DMG_CSFL7_3D	HGG_T8	DMG_CSFE1_3D	HGG_T4	LGG_(T1…T45)	MB_CSFE4_2D	MB_CSL2_2D	MNG_T4
	MB_D283_ST63_2D-3D	MB_G3_O73	MB_G4_T7	MB_G3_T42	MB_G3_T1	MB_WNT_T6	MB_SHHb_T60	EPN_T5	HGG_T3	DMG_CSFL13c_3D	HGG_T25	DMG_CSFE6b_3D	HGG_T18	LGG_CSE3_2D	LGG_CSL5_2D	MB_CSE2_2D	MNG_T3
	MB_CHLA_CSFL62	MB_G3_O72	MB_G4_T5	MB_G3_T41	MB_CSFL1b_3D	MB_WNT_T58	MB_SHHb_T52	EPN_T4	HGG_T1	DMG_CSFL13b_2D	HGG_T24	DMG_CSFE6a_3D	HGG_T11	LGG_CSE1a_2D	LGG_CSFE14_2D	LGG_CSL3_2D	MNG_T2
	MB_DAOY_ST65_2D	MB_G3_O71	MB_G4_T45	MB_G3_T40	MB_CSFL1a_3D	MB_WNT_T57	MB_SHHb_T51	EPN_T3	HGG_CSFE5b_3D	DMG_CSFL13_3D	HGG_T16	DMG_CSFE6a_2D	DMG_T5	LGG_CSE11_2D	HGG_CSFL2_3D	LGG_CSL11b_2D	MNG_T1
	MB_CSFL3b_3D	MB_G3_O70	MB_G4_T44	MB_G3_T39		MB_WNT_T50	MB_SHHb_T36	EPN_T2	HGG_CSFE5a_3D	DMG_CSFL9b_2D	HGG_T15	DMG_CSFE1_2D	DMG_T24	HGG_T7	HGG_CSFE4b_2D	LGG_CSL11a_2D	
	MB_CSFL3a_3D	MB_G3_O69	MB_G4_T43	MB_G3_T32		MB_WNT_T49	MB_SHHb_T35	EPN_T1	HGG_CSFE3_3D	DMG_CSFL7_2D	HGG_T14		DMG_T23	HGG_T6	HGG_CSFE4a_2D	LGG_CSL9_2D	
	LGG_CSL4_2D	MB_G3_O68	MB_G4_T38	MB_G3_T28		MB_WNT_T48	MB_SHHb_T3	EPN_CSFE2_2D	HGG_CSFE1b_3D	DMG_CSFL4_2D	HGG_CSFL8c_2D		DMG_T20	HGG_T23	HGG_CSFE2_3D	LGG_CSL8_2D	
	LGG_CSE4_2D	MB_G3_O67	MB_G4_T37	MB_G3_T24		MB_WNT_T47	MB_SHHb_T23	EPN_CSFE1_2D	HGG_CSFE1a_2D	DMG_CSFL13_2D	DMG_T4		DMG_T18	HGG_T22	DMG_CSFE15_2D	LGG_CSL7_2D	
	HGG_T19	MB_G3_O66	MB_G4_T34	MB_G3_T16		MB_WNT_T46	MB_SHHa_T59			DMG_CSFE8a_2D			DMG_T1	HGG_T21	DMG_CSFL2_2D	LGG_CSL6_2D	
	HGG_KNS42_10_2D		MB_G4_T31	MB_G3_T12		MB_WNT_T11	MB_SHHa_T56			DMG_CSFE12_2D			DMG_CSFE16_2D	HGG_T20	DMG_CSFE5b_2D	LGG_CSL2_2D	
	HGG_CSFL9_2D		MB_G4_T30	MB_G3_T10			MB_SHHa_T55							HGG_T2	DMG_CSFE5a_2D	LGG_CSL13_2D	
	HGG_CSFL6_3D		MB_G4_T29				MB_SHHa_T54							HGG_T17	DMG_CSFE17_2D	LGG_CSL12_2D	
	DMG_T21		MB_G4_T26				MB_SHHa_T53							HGG_T13	DMG_CSFE10_2D	LGG_CSL10b_2D	
	DMG_CSFE11_2D		MB_G4_T25				MB_SHHa_T4							HGG_T12		LGG_CSL10a_2D	
	DMG_CSFE3_2D		MB_G4_T21				MB_SHHa_T27							HGG_CSFE7_2D		LGG_CSFL9_3D	
			MB_G4_T19				MB_SHHa_T22							HGG_CSFE6_3D		LGG_CSFL12_3D	
			MB_G4_T18				MB_SHHa_T2							EPN_T9		LGG_CSFE13_3D	
			MB_G4_T17											EPN_T8		HGG_CSFL8b_3D	
			MB_G4_T15											EPN_T7		HGG_CSFL8a_3D	
			MB_G4_T13											DMG_T3b			
			MB_G3_T33											DMG_T3a			
			MB_CSFE5_2D											DMG_T22			
														DMG_T2			
														DMG_T19			
Validity index	0.92	0.88	0.26	0.26	0.51	0.82	0.86	0.9	0.85	0.66	0.75	0.87	0.29	0.59	0.8	0.38	0.8

Supplementary Table 5. The unsupervised assignment of samples to clusters resulting from HDBSCAN density-based clustering method. Validity indexes computed by HDBSCAN (DBCV score).

Supplementary Table 6. Genome-wide differentially methylated regions (DMRs) in pediatric brain tumors (pBTs) and cell lines.

DMR analysis	Reference group	Test group	# of DMRs
DMR cluster analysis 1 (DCA1)	(clusters #9, 12, 13) DMG_CSFE11_2D, DMG_CSFL4_2D DMG_T1, DMG_T2, DMG_T3a, DMG_T3b, DMG_T4, DMG_T5, DMG_T18, DMG_T19, DMG_T20, DMG_T22, DMG_T23, DMG_T24, EPN_T7, EPN_T8, EPN_T9, HGG_CSFE6_3D, HGG_CSFE7_2D, HGG_CSFL8c_2D, HGG_T2, HGG_T4, HGG_T6, HGG_T7, HGG_T8, HGG_T11, HGG_T12, HGG_T13, HGG_T14, HGG_T15, HGG_T16, HGG_T17, HGG_T18, HGG_T20, HGG_T21, HGG_T22, HGG_T23, HGG_T24, HGG_T25, LGG_CSE1a_2D, LGG_CSE3_2D, LGG_CSE11_2D, LGG_T1, LGG_T2, LGG_T3, LGG_T4, LGG_T5, LGG_T6, LGG_T7, LGG_T8, LGG_T9, LGG_T11, LGG_T12, LGG_T13, LGG_T14, LGG_T15a, LGG_T15b, LGG_T16, LGG_T17, LGG_T18, LGG_T19, LGG_T20a, LGG_T20b, LGG_T21, LGG_T22, LGG_T23, LGG_T24, LGG_T25, LGG_T26, LGG_T27, LGG_T28, LGG_T29, LGG_T30, LGG_T31, LGG_T32, LGG_T33, LGG_T34, LGG_T35, LGG_T36, LGG_T37, LGG_T38, LGG_T39, LGG_T40, LGG_T41, LGG_T42, LGG_T43, LGG_T45	(clusters #14, 15) LGG_CSL3_2D, DMG_CSFE10_2D, DMG_CSFE15_2D, DMG_CSFE17_2D, DMG_CSFE5a_2D, DMG_CSFE5b_2D, DMG_CSFL2_2D, HGG_CSFE2_3D, HGG_CSFE4a_2D, HGG_CSFE4b_2D, HGG_CSFL2_3D, HGG_CSFL8a_3D, HGG_CSFL8b_3D, LGG_CSFE13_3D, LGG_CSFE14_2D, LGG_CSFL12_3D, LGG_CSFL9_3D, LGG_CSL10a_2D, LGG_CSL10b_2D, LGG_CSL11a_2D, LGG_CSL11b_2D, LGG_CSL12_2D, LGG_CSL13_2D, LGG_CSL2_2D, LGG_CSL5_2D, LGG_CSL6_2D, LGG_CSL7_2D, LGG_CSL8_2D, LGG_CSL9_2D	13,238
DMR cluster analysis 2 (DCA2)	(cluster #15) HGG_CSFL8a_3D, HGG_CSFL8b_3D, LGG_CSFE13_3D, LGG_CSFL12_3D, LGG_CSFL9_3D, LGG_CSL10a_2D, LGG_CSL10b_2D, LGG_CSL11a_2D, LGG_CSL11b_2D, LGG_CSL12_2D, LGG_CSL13_2D, LGG_CSL2_2D, LGG_CSL3_2D, LGG_CSL6_2D, LGG_CSL7_2D, LGG_CSL8_2D, LGG_CSL9_2D, MB_CSE2_2D, MB_CSL2_2D	(cluster #14) MB_CSFE4_2D, LGG_CSL5_2D, LGG_CSFE14_2D, HGG_CSFL2_3D, HGG_CSFE4b_2D, HGG_CSFE4a_2D, HGG_CSFE2_3D, DMG_CSFE15_2D, DMG_CSFL2_2D, DMG_CSFE5b_2D, DMG_CSFE5a_2D, DMG_CSFE17_2D, DMG_CSFE10_2D	10,477
MB	MB_D341_ST64_3D, MB_D283_ST63_2D-3D, MB_CSFL1b_3D, MB_CSFL1a_3D, MB_CSFE5_2D, MB_CHLA_CSFL6	MB_DAOY_ST65_2D, MB_CSL2_2D, MB_CSFL3b_3D, MB_CSFL3a_3D, MB_CSFE4_2D, MB_CSE2_2D	1,387
LGG	LGG_T2, LGG_T3, LGG_T4, LGG_T5, LGG_T6, LGG_T7, LGG_T8, LGG_T9, LGG_T11, LGG_T12, LGG_T13, LGG_T14, LGG_CSE3_2D, LGG_CSE1a_2D, LGG_CSE11_2D	LGG_CSL2_2D, LGG_CSL3_2D, LGG_CSL4_2D, LGG_CSE4_2D, LGG_CSL5_2D, LGG_CSL6_2D, LGG_CSL7_2D, LGG_CSL8_2D, LGG_CSL9_2D, LGG_CSFL9_3D, LGG_CSL10a_2D, LGG_CSL10b_2D, LGG_CSL11a_2D, LGG_CSL11b_2D, LGG_CSL12_2D, LGG_CSL13_2D, LGG_CSFL12_3D, LGG_CSFE13_3D, LGG_CSFE14_2D	9,666

HGG	HGG_CSFE1a_2D, HGG_CSFE1b_3D, HGG_CSFE3_3D, HGG_CSFE5a_3D, HGG_CSFE5b_3D, HGG_CSFE6_3D, HGG_CSFL6_3D, HGG_CSFE7_2D, HGG_CSFL8c_2D, HGG_CSFL9_2D, HGG_KNS42_10_2D	HGG_CSFE2_3D, HGG_CSFL2_3D, HGG_CSFE4b_2D, HGG_CSFE4a_2D, HGG_CSFL8b_3D, HGG_CSFL8a_3D	5,009
DMG	DMG_CSFE1_2D, DMG_CSFL4_2D, DMG_CSFE6a_2D, DMG_CSFL7_2D, DMG_CSFE8a_2D, DMG_CSFL9b_2D, DMG_CSFE11_2D, DMG_CSFE12_2D, DMG_CSFL13_2D, DMG_CSFE16_2D	DMG_CSFL2_2D, DMG_CSFE3_2D, DMG_CSFE5b_2D, DMG_CSFE5a_2D, DMG_CSFE10_2D, DMG_CSFE15_2D, DMG_CSFE17_2D	5,576

Supplementary Table 6. **Sample set compositions of pediatric brain tumors (pBTs) and cell lines used to perform the 7 DMR analysis.**

Supplementary Table 7. Summary of the enriched cell pathways/processes obtained by analysis of DMRs by means of missMethyl (analysis 1) and the additional enrichment analysis of hypo -hypermethylated genes performed with clusterProfiler (analysis 2)

	DMR cluster analysis 1 (DCA1)	DMR cluster analysis 2 (DCA2)	MB	LGG	HGG	DMG
enrichment analysis 1[1]:						
# of KEGG pathways	-	-	-	29[2]	-	1[3]
# of MSigDB Hallmark gene sets	32	25	5	24	7	1
# of Gene Ontology terms / # of genes involved	998/9,455	532/8,379	12/520	806/5,346	95/2,778	198/3,137
# of hypo-/hyper- methylated genes[4]	308/396	752/171	351/66	611/764	866/578	331/104
enrichment analysis 2:						
# of Gene Ontology terms / # of hypomethylated genes	41/84	246/467	539/284[5]	92/259[5]	159/506[5]	150/177[5]
# of Gene Ontology terms / # of hypermethylated genes	129/207	304/92	66/46	388/376	10/79	21/21

[1] restricted to DMRs which include promoters.

[2] MicroRNAs in cancer, Cytokine-cytokine receptor interaction, PI3K-Akt signaling pathway, Pertussis, Pathways in cancer, Ether lipid metabolism, PPAR signaling pathway, Neuroactive ligand-receptor interaction, Th17 cell differentiation, Ras signaling pathway, Fatty acid metabolism, Th1 and Th2 cell differentiation, Malaria, Glycerophospholipid metabolism, AGE-RAGE signaling pathway in diabetic complications, TNF signaling pathway, Inflammatory bowel disease, Apelin signaling pathway, cAMP signaling pathway, Hematopoietic cell lineage, Chemokine signaling pathway, Metabolic pathways, Hypertrophic cardiomyopathy, ECM-receptor interaction, Glycosphingolipid biosynthesis - lacto and neolacto series, Leukocyte transendothelial migration, IL-17 signaling pathway, Proteoglycans in cancer, ToxoplasmosisMicroRNAs in cancer, Cytokine-cytokine receptor interaction, PI3K-Akt signaling pathway, Pertussis, Pathways in cancer, Ether lipid metabolism, PPAR signaling pathway, Neuroactive ligand-receptor interaction, Th17 cell differentiation, Ras signaling pathway, Fatty acid metabolism, Th1 and Th2 cell differentiation, Malaria, Glycerophospholipid metabolism, AGE-RAGE signaling pathway in diabetic complications, TNF signaling pathway, Inflammatory bowel disease, Apelin signaling pathway, cAMP signaling pathway, Hematopoietic cell lineage, Chemokine signaling pathway, Metabolic pathways, Hypertrophic cardiomyopathy, ECM-receptor interaction, Glycosphingolipid biosynthesis - lacto and neolacto series, Leukocyte transendothelial migration, IL-17 signaling pathway, Proteoglycans in cancer, Toxoplasmosis.

[3] Neuroactive ligand-receptor interaction.

[4] $\Delta\beta$ +/- 10%. These gene lists were used to perform the enrichment analysis 2 by means of clusterProfiler.

[5] The overlap among these hypomethylated gene sets is reported as venn diagram in Fig. 6A.

Supplementary Table 7. Summary of the number of enriched cell pathways/processes obtained by analysis of DMRs by means of missMethyl (analysis 1) and the additional enrichment analysis of hypo-/hypermethylated genes performed with clusterProfiler.

The total amount of significant hypo-/ hypermethylated genes associated with the Gene Ontology processes is also reported.

Supplementary Table 8. Enrichment analyses for genome-wide differentially methylated regions (DMRs) in pediatric brain tumors (pBTs) and cell lines.

Enrichment analysis	Significantly enriched Hallmark gene sets (MSigDB)
DMR cluster analysis 1 (DCA1)*	TNFA_SIGNALING_VIA_NFKB, HYPOXIA, EPITHELIAL_MESENCHYMAL_TRANSITION, ESTROGEN_RESPONSE_EARLY, MYOGENESIS, ESTROGEN_RESPONSE_LATE, APOPTOSIS, GLYCOLYSIS, INFLAMMATORY_RESPONSE, P53_PATHWAY, UV_RESPONSE_DN, IL2_STAT5_SIGNALING, APICAL_JUNCTION, MTORC1_SIGNALING, INTERFERON_GAMMA_RESPONSE, FATTY_ACID_METABOLISM, WNT_BETA_CATENIN_SIGNALING, IL6_JAK_STAT3_SIGNALING, KRAS_SIGNALING_UP, TGF_BETA_SIGNALING, COMPLEMENT, HEME_METABOLISM, ADIPOGENESIS, E2F_TARGETS, UV_RESPONSE_UP, PEROXISOME, CHOLESTEROL_HOMEOSTASIS, SPERMATOGENESIS, REACTIVE_OXYGEN_SPECIES_PATHWAY, COAGULATION, XENOBIOTIC_METABOLISM, INTERFERON_ALPHA_RESPONSEiTNFA_SIGNALING_VIA_NFKB, HYPOXIA, EPITHELIAL_MESENCHYMAL_TRANSITION, ESTROGEN_RESPONSE_EARLY, MYOGENESIS, ESTROGEN_RESPONSE_LATE, APOPTOSIS, GLYCOLYSIS, INFLAMMATORY_RESPONSE, P53_PATHWAY, UV_RESPONSE_DN, IL2_STAT5_SIGNALING, APICAL_JUNCTION, MTORC1_SIGNALING, INTERFERON_GAMMA_RESPONSE, FATTY_ACID_METABOLISM, WNT_BETA_CATENIN_SIGNALING, IL6_JAK_STAT3_SIGNALING, KRAS_SIGNALING_UP, TGF_BETA_SIGNALING, COMPLEMENT, HEME_METABOLISM, ADIPOGENESIS, E2F_TARGETS, UV_RESPONSE_UP, PEROXISOME, CHOLESTEROL_HOMEOSTASIS, SPERMATOGENESIS, REACTIVE_OXYGEN_SPECIES_PATHWAY, COAGULATION, XENOBIOTIC_METABOLISM, INTERFERON_ALPHA_RESPONSE
DMR cluster analysis 2 (DCA2)**	MTORC1_SIGNALING, MYC_TARGETS_V1, DNA_REPAIR, XENOBIOTIC_METABOLISM, E2F_TARGETS, IL2_STAT5_SIGNALING, MYOGENESIS, OXIDATIVE_PHOSPHORYLATION, UNFOLDED_PROTEIN_RESPONSE, ANDROGEN_RESPONSE, MYC_TARGETS_V2, TNFA_SIGNALING_VIA_NFKB, HYPOXIA, P53_PATHWAY, UV_RESPONSE_DN, SPERMATOGENESIS, GLYCOLYSIS, UV_RESPONSE_UP, ESTRO GEN_RESPONSE_LATE, G2M_CHECKPOINT, ESTROGEN_RESPONSE_EARLY, MITOTIC_SPINDLE, REACTIVE_OXYGEN_SPECIES_PATHWAY, ADIPOGENESIS, COMPLEMENT, APOPTOSIS, APICAL_JUNCTION, HEME_METABOLISM, PI3K_AKT_MTOR_SIGNALING, CHOLESTEROL_HOMEOSTASIS, INTERFERON_GAMMA_RESPONSE, NOTCH_SIGNALING, INTERFERON_ALPHA_RESPONSE, FATTY_ACID_META BOLISM, KRAS_SIGNALING_UP, INFLAMMATORY_RESPONSE, PEROXISOME, EPITHELIAL_MESENCHYMAL_TRANSITION, TGF_BETA_SIGNALING, WNT_BETA_CATENIN_SIGNALING, PROTEIN_SECRETION, HEDGEHOG_SIGNALING, IL6_JAK_STAT3_SIGNALING, APICAL_SURFACE, ANGIOGENESIS
MB	EPITHELIAL_MESENCHYMAL_TRANSITION, APOPTOSIS, INTERFERON_GAMMA_RESPONSE, INTERFERON_ALPHA_RESPONSE, KRAS_SIGNALING_UP
LGG	EPITHELIAL_MESENCHYMAL_TRANSITION, HYPOXIA, MYOGENESIS, TNFA_SIGNALING_VIA_NFKB, INFLAMMATORY_RESPONSE, ESTROGEN_RESPONSE_LATE, ESTROGEN_RESPONSE_EARLY, GLYCOLYSIS, APOPTOSIS, IL6_JAK_STAT3_SIGNALING, APICAL_JUNCTION, INTERFERON_GAMMA_RESPONSE, IL2_STAT5_SIGNALING, COMPLEMENT, P53_PATHWAY, COAGULATION, UV_RESPONSE_DN, ALLOGRAFT_REJECTION, WNT_BETA_CATENIN_SIGNALING, ANGIOGENESIS, INTERFERON_ALPHA_RESPONSE, XENOBIOTIC_METABOLISM, UV_RESPONSE_UP, APICAL_SURFACE
HGG	MYOGENESIS, ESTROGEN_RESPONSE_LATE, COAGULATION, KRAS_SIGNALING_DN, EPITHELIAL_MESENCHYMAL_TRANSITION, APICAL_JUNCTION, XENOBIOTIC_METABOLISM, APOPTOSIS, IL2_STAT5_SIGNALING, SPERMATOGENESIS, CHOLESTEROL_HOMEOSTASIS, PANCREAS_BETA_CELLS, ESTROGEN_RESPONSE_EARLY, HYPOXIA, KRAS_SIGNALING_UP, REACTIVE_OXYGEN_SPECIES_PATHWAY, INTERFERON_GAMMA_RESPONSE, INFLAMMATORY_RESPONSE, PEROXISOME, TGF_BETA_SIGNALING, COMPLEMENT, BILE_ACID_METABOLISM
DMG	MYOGENESIS, INFLAMMATORY_RESPONSE, KRAS_SIGNALING_DN, PANCREAS_BETA_CELLS, ESTROGEN_RESPONSE_EARLY, EPITHELIAL_MESENCHYMAL_TRANSITION, HYPOXIA, APICAL_JUNCTION, COAGULATION, KRAS_SIGNALING_UP, INTERFERON_ALPHA_RESPONSE, SPERMATOGENESIS, PEROXISOME, XENOBIOTIC_METABOLISM, ESTROGEN_RESPONSE_LATE, COMPLEMENT, UV_RESPONSE_UP, INTERFERON_GAMMA_RESPONSE, APOPTOSIS

* DCA1: molecular differences between the unfaithful cells (clusters #14 and #15) vs tumor samples and the respective faithful derived cell lines (mainly part of clusters #9, #12, #13) (See Supplementary Table 6 and Supplementary Figure 3 for details).
** DCA2: molecular differences between unfaithful serum free-2D cell lines (cluster #14) and meningioma-like cells (cluster #15) (See Supplementary Table 6and Supplementary Figure 3 for details).

Supplementary Table 8. **Lists of significant enriched Hallmark gene sets (MSigDB) detected by *gsaregion* (missMethyl).**
